# Effects of Ramadan and Non-ramadan Intermittent Fasting on Body Composition: A Systematic Review and Meta-Analysis

**DOI:** 10.3389/fnut.2020.625240

**Published:** 2021-01-26

**Authors:** Joana M. Correia, Inês Santos, Pedro Pezarat-Correia, Analiza M. Silva, Goncalo V. Mendonca

**Affiliations:** ^1^Neuromuscular Research Lab, Faculty of Human Kinetics, University of Lisbon, Lisbon, Portugal; ^2^Centro Interdisciplinar para o Estudo da Performance Humana (CIPER), Faculty of Human Kinetics, University of Lisbon, Lisbon, Portugal; ^3^Centro de Investigação em Desporto, Educação Física, Exercício e Saúde (CIDEFES), Lusófona University, Lisbon, Portugal; ^4^Nutrition Lab, Faculty of Medicine, University of Lisbon, Lisbon, Portugal; ^5^Exercise and Health Laboratory, Faculty of Human Kinetics, University of Lisbon, Lisbon, Portugal

**Keywords:** Energy restriction, nutrition, diet, weight loss, calorie, exercise

## Abstract

Intermittent fasting (IF) has gained popularity for body-composition improvement purposes. The aim of this systematic review and meta-analysis was to summarize the effects of Ramadan vs. non-Ramadan IF on parameters of body composition. We conducted a comprehensive search of peer-reviewed articles in three electronic databases: PubMed, Scopus, and Web of Science (published until May 2020). Studies were selected if they included samples of adults (≥18 years), had an experimental or observational design, investigated any type of IF and included body composition outcomes. Meta-analytical procedures were conducted when feasible. Sixty-six articles met the eligibility criteria. We found that non-Ramadan IF is effective for decreasing body weight (−0.341 (95% CI [−0.584, −0.098], *p* = 0.006), body mass index (−0.699, 95% CI [−1.05, −0.347], *p* < 0.001), and absolute fat mass (−0.447, 95% CI [−0.673, −0.221], *p* < 0.001). When contrasting pre- post-intervention data on fat-free mass between treatments and controls, group-differences were non-significant (*p* > 0.05). Conversely, we observed a significant increase in fat-free mass when comparing pre- to post-intervention in a within design fashion (0.306, 95% CI [0.133, 0.48], *p* = 0.001). Finally, despite being accompanied by dehydration, Ramadan IF is effective in decreasing body weight (−0.353; 95% CI [−0.651, −0.054], *p* = 0.02) and relative fat mass (−0.533; 95% CI [−1.025, −0.04], *p* = 0.034). Ramadan IF seems to implicate some beneficial adaptations in weight management, although non-Ramadan IF appears to be more effective in improving overall body composition.

## Key Points

- Both Ramadan and non-Ramadan intermittent fasting are effective on fat mass and body weight losses.
- Fat mass loss is more pronounced with non-Ramadan intermittent fasting and this type of intermittent fasting, combined with exercise training, leads to higher decreases in body mass index.

- Non-Ramadan intermittent fasting may be well-suited for eliciting small increases in fat-free mass, particularly under circumstances involving the simultaneous control of caloric intake.

## Introduction

There are several forms of intermittent fasting (IF), all using fasting periods that extend well-beyond the duration of an overnight fast and implicating limited feeding time-windows, with or without caloric restriction (1, 2). For example, in time-restricted feeding/eating (TRF), eating is limited to a certain number of hours each day (3). Alternate-day fasting (ADF) consists of alternating between feasting and fasting days—feasting is compatible with *ad libitum* energy intake during 24 h and fasting implicates a caloric intake ≤ 25% of daily needs (~ 500/600 kcal during 24 h) (4, 5). Recently, a focus on intermittent energy restriction (IER) has emerged in the scientific literature as an alternative approach to continuous energy restriction (CER) for improving body composition (6). It includes periods of energy restriction alternated with periods of habitual intake or minimally restricted dietary intake, allowing wider food choices (6, 7). Various forms of IER are currently being used for clinical purposes, including the “5:2 diet” and the “week-on-week-off” (7–9).

The ultimate goal of most IF regimens is to improve body composition. For this reason, they are suggested to maximize the loss of fat mass (FM), while attempting to preserve fat-free mass (FFM) (3). There is compelling evidence that IF elicits reductions in body weight and FM of ~3–8% (4, 10–14) and ~4–15%, respectively (1, 4, 10–13). Improvements in blood lipid profile, blood pressure and insulin sensitivity have also been consistently reported (5, 14). While the majority of studies explored the effectiveness of IF on improving body composition in adults with overweight/obesity (4, 5, 11, 13–15), there are only occasional reports that included normoponderal individuals (10, 13). Despite not being a universal finding (2, 16), IF has also been shown to induce slight increases in FFM [e.g., (17)]. In this specific study, the authors used a crossover design to compare TRF with an alternative diet involving three meals per day for a period of 8 weeks, with energy intake being individualized to each participant to ensure maintenance of body weight throughout both treatments (TRF and non-TRF). Under these conditions, TRF was shown to be effective in decreasing FM and increasing FFM (by ~1.5 kg; *p* = 0.06) (17). In what concerns IER, and contrary to the initial belief, it seems to be unrelated to compensatory hyperphagia on *ad libitum* days; thus, similarly to that seen during CER, IER is accompanied by a negative caloric balance and this results in comparable changes in body composition between both interventions after 12 months of treatment (7). However, instead of being externally driven, caloric restriction during IER is purely spontaneous (18).

Apart from being used for clinical purposes, IF is also used in many religious practices. A notable example is Ramadan IF, which is the most widely-studied form of IF (19). During Ramadan, Muslims abstain from ingesting food and liquids between sunrise and sunset throughout a month-long period (20, 21). Thus, food and liquid intake becomes exclusively nocturnal. Even though Muslims eat *ad libitum* after sunset and before dawn, caloric restriction often accompanies Ramadan IF (22). Ramadan IF can last ~12–18 h/day, depending on the geographic location and season of the year (23–25). Reductions in body weight, relative FM and resting metabolic rate are common consequences of Ramadan IF (20, 26). It has also been shown that Ramadan-related caloric restriction decreases total cholesterol, low-density lipoprotein (LDL) and fasting blood glucose levels. In parallel, Ramadan IF seems to improve body composition, possibly through enhancements in the ability to mobilize saturated fatty acids for metabolic processes (25). Changes in body composition may also reflect a balance between the unavoidable decrease of meal frequency (i.e., from 3–4 to 2) and sleep duration, together with a reduction of spontaneous daily physical activity and coercive dehydration (27–29). However, the effects of Ramadan IF on body composition are not conclusive, with some studies indicating significant reductions in body weight (19, 20, 23, 24, 26, 28, 30–34) and many other failing to show any relevant changes (21, 22, 27, 35–40). Such inconsistencies might be secondary to the specificities inherent to the participants included in each study. Alternatively, they may be dependent on differences between studies at the level of macro and micronutrient intake, or even arise as a consequence of cultural rituals or the number of fasting hours (23).

Taking all these points into consideration, this systematic review and meta-analysis aimed at summarizing the existing evidence on the effect of various IF regimens (i.e., TRF, ADF, IER, and Ramadan IF) on body composition. In addition, we sought to determine whether Ramadan and non-Ramadan IF exert a differential impact on human body composition. We hypothesized that various types of IF might contribute for improving body composition and that this effect might be further enhanced in circumstances compatible with a non-Ramadan dietary approach. To our knowledge, this is the first study providing such a comprehensive perspective.

## Methods

This systematic review and meta-analysis was conducted in accordance with the Preferred Reporting Items for Systematic Reviews and Meta-Analysis (PRISMA) statement (41) and is registered on PROSPERO (registration number CRD42020191161).

### Eligibility Criteria

Articles focusing on the interaction between IF and body composition outcomes were retrieved. Only original research published in peer-reviewed scientific journals were considered. Studies were selected for this review if they respected the following criteria: (i) included samples of adults (≥18 years), regardless of sex, (ii) had an experimental or observational design, (iii) investigated any type of IF (i.e., TRF, ADF, IER, or Ramadan IF), and (iv) included body composition outcomes (i.e., body weight, FFM, FM, muscle mass/volume, bone density, waist circumference, or body mass index—BMI). Studies including persons with specific health conditions, taking medication or having diseases/conditions known to affect metabolism/body composition (e.g., cancer, thyroid disease, diabetes, bariatric surgery, total parenteral nutrition, human immunodeficiency virus/acquired immunodeficiency syndrome (HIV/AIDS), organ transplant, Prader–Willi Syndrome, polycystic ovary syndrome, chronic obstructive pulmonary disease, or acute illnesses, such as infections or traumatic injury), or pregnant women (or breastfeeding) were not included. Review articles, case studies, protocols, as well as abstracts/conference papers were also excluded.

### Search Strategy

A comprehensive search of peer-reviewed articles was conducted in May 2020 in the following electronic databases: PubMed, Scopus and Web of Science. Searches included combinations of two sets of terms: (i) terms concerning the dietary intervention (i.e., IF) and (ii) terms representing the outcome of interest (i.e., body composition). Free-text terms used for the literature search were as follows (based on keywords): (“intermittent fasting” OR “alternate-day-fasting” OR “time-restricted feeding” OR “time-restricted eating” OR “periodic fasting” OR “intermittent calorie restriction” OR “intermittent energy restriction” OR “fasted state” OR “Ramadan”) AND (“body composition” OR “body mass” OR “body mass index” OR “Quetelet index” OR “fat-free mass (FFM)” OR “fat-free mass” OR “fat mass” OR “fat percentage” OR “body weight” OR “waist circumference” OR “muscle mass” OR “muscle volume” OR “bone density”). There were no restrictions regarding the language of publication. Additionally, manual cross-referencing of literature cited in previous key reviews were performed.

### Screening and Data Extraction

All titles and abstracts identified from the literature searches were screened for potential inclusion eligibility by three authors (JC, IS and GVM), based on the eligibility criteria mentioned above. Duplicate entries were removed. Relevant studies were fully screened by the same authors. A data extraction form was developed to compile information about the article (i.e., authors and year of publication), participants (i.e., sample size and demographics), study design, intervention characteristics (i.e., methods, protocols and length of intervention), and outcomes of interest. Endnote® X7 for Windows 10 was used to manage the references. The first author (JC) extracted the data and uncertainties were resolved by consensus.

### Quality Assessment

Study quality was assessed with the Quality Assessment Tool for Quantitative Studies developed by the Effective Public Health Practice Project (EPHPP) (42), evaluating five key methodological domains: selection bias, study design, confounders, data collection method and withdrawals/dropouts. Since it is not possible to blind participants regarding a fasting condition, the sixth item of the original scale (blinding) was not assessed. Each domain was classified as strong, moderate or weak methodological quality. A global rating was determined based on the scores of each component. The five domains and overall quality were rated independently by two authors (JC and IS), and discrepancies were discussed until a consensus was reached. Inter-rater agreement across categories varied from moderate (Cohen's *k* = 0.6) to strong (*k* = 1).

### Data Synthesis

For each study, results were summarized by: (i) intervention characteristics (intervention protocols, methods and duration), (ii) changes in body weight, (iii) changes in BMI, (iv) changes in absolute and relative FM, and (v) changes in FFM.

### Data Analysis

Analyses were conducted using the Comprehensive Meta-Analysis (CMA) software version 3.0 (43). Separate meta-analyses were performed for each outcome (BMI, FM, FFM, and body weight) considering all studies, only Ramadan IF and only non-Ramadan IF studies. Meta-analyses were conducted using both random-effects and fixed-effect models, in which the summary effect size (ES) is the standardized mean difference (SMD) of a distribution (44, 45). Random-effects models were chosen for most analyses due to the high number of available studies (*k* ≥ 6), assuming that the true effect size varies from study to study and that the studies in our analysis represent a random sample of effect sizes. When the number of studies per outcome was limited (*k* < 6), we used fixed-effect models, assuming that the true effect size is the same in all studies and the only reason for the effect size to vary between studies is sampling error (44). SMDs were calculated based on sample size, standard differences in means (between pre- and post-intervention time points and between pre- and post-intervention data of both treatment and control groups) and effect direction, and were interpreted according to Cohen's guidelines, with values of 0.2, 0.5 and 0.8 for small, medium and large SMD, respectively (46). The 95% confidence interval (CI) and corresponding *p*-values were considered as indicators of statistical significance. To evaluate the amount of variation in the effects of included studies, we tested for heterogeneity using the following approach: (i) the Cochran's Q statistic (47), for which a significant *p*-value (< 0.05) demonstrates that studies do not share a common SMD (i.e., there is heterogeneity in the effect sizes between studies); and (ii) the *I*^2^ statistic (48) that assesses the proportion of observed dispersion that is due to real differences in the actual SMD and is not affected by low statistical power. The *I*^2^ ranges from 0 to 100%, where a value of 0% indicates no observed heterogeneity and values of 25, 50, and 75% reflect low, moderate and high heterogeneity, respectively (48). Some studies did not provide sufficient data to estimate the SMD and therefore the number of studies per outcome reported in the qualitative synthesis may differ from the ones included in the meta-analyses.

Subgroup analyses were conducted to examine whether the effect of IF on body composition outcomes varied according to the pre-intervention nutritional status (overweight/obesity vs. normal body weight), exercise (protocols with vs. without exercise), and caloric intake (controlling for vs. not controlling for caloric intake). These subgroup analyses were conducted using mixed-effect models (i.e., random-effects model was conducted within subgroups and a fixed-effect model was used across subgroups) (49). Between-groups Q statistic and corresponding *p*-values were used to compare the mean effect across subgroups. Some studies included samples with both individuals showing normal body weight and excess weight/obesity, and therefore could not be included in moderation analyses.

### Sensitivity Analyses

Sensitivity analyses were carried out to explore if the results were affected by methodological quality. Therefore, primary analyses were repeated excluding studies with poor methodological quality. Publication bias was also examined by visual inspection of funnel plots for asymmetry. To quantitatively confirm the visual impression, Egger's test (50) and Duval and Tweedie's trim-and-fill method (51) were used, but only for meta-analyses with 10 or more studies per outcome and no substantial heterogeneity, since the power is too low to distinguish chance from real asymmetry.

## Results

### Study Selection

**Figure 1:**
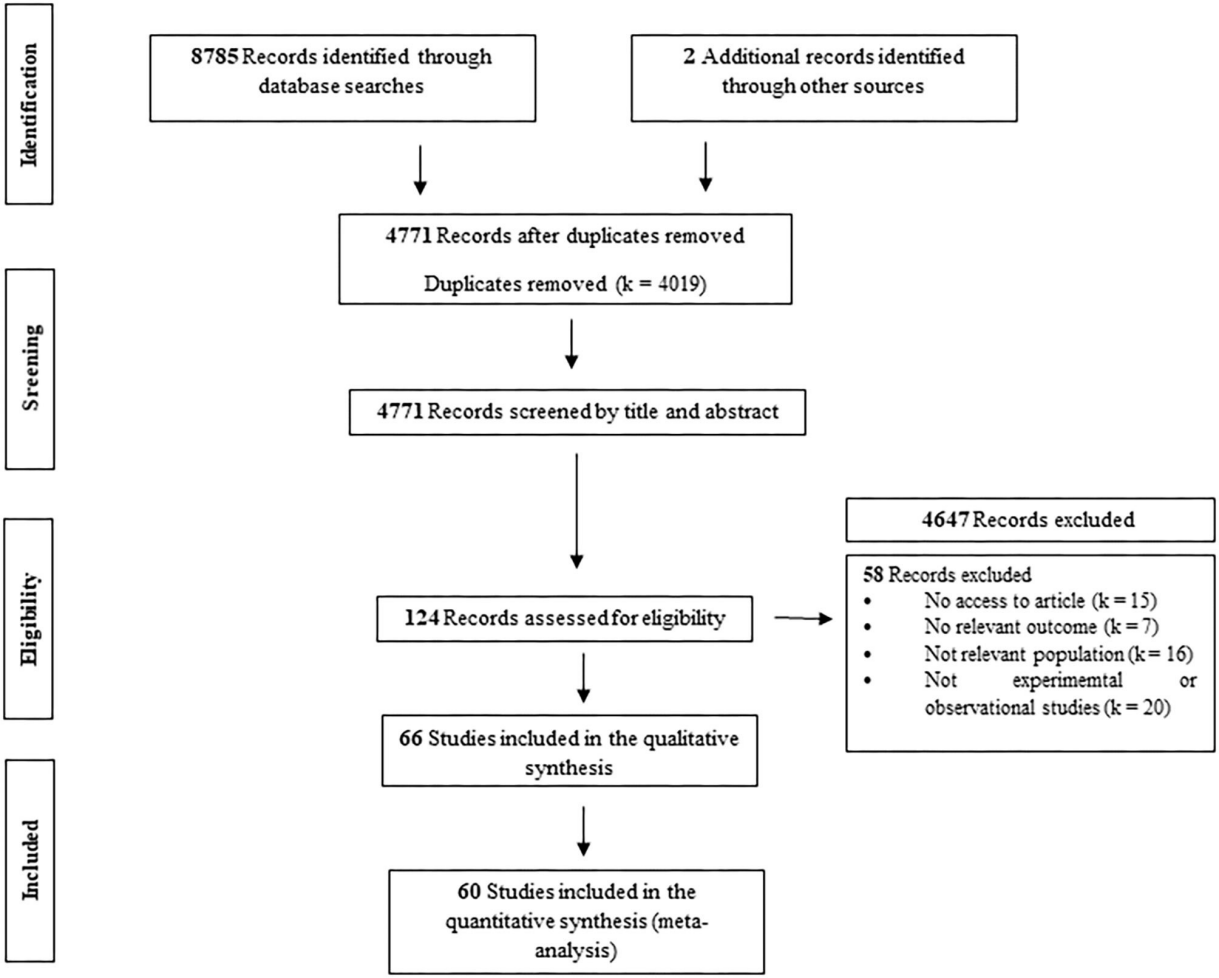
Flow chart of the methodology for the search results.

PubMed, Scopus and Web of Science searches generated 8,785 publications. Two publications were manually added from previous key reviews. Of 4,771 articles (after duplicates were removed), 124 were considered potentially relevant. Sixty-six articles met all inclusion criteria and were included in the present review (Figure 1). Of these, 60 provided sufficient data to be included in the meta-analyses.

### Study Characteristics

Characteristics of included studies are summarized in Supplementary Table 1. Twenty-seven studies were randomized controlled trials, 13 studies were non-randomized controlled trials, four studies were quasi-experimental (i.e., used a single-group pre- post-test design, where participants were their own controls) and 22 were observational studies. All IF protocols were conducted in laboratories (*k* = 66). More than half were undertaken during the month of Ramadan (*k* = 34) and the others explored the effects of TRF vs. normal diet on body composition (*k* = 12), during a period of 12 weeks (*k* = 3), 8 weeks (*k* = 5), 6 weeks (*k* = 2), 4 weeks (*k* = 1), and 10 days (*k* = 1); ADF vs. CER (*k* = 4) during 24 weeks (*k* = 2), 16 weeks (*k* = 1), and 8 weeks (*k* =1), or ADF vs. no diet (*k* = 8) during 24 weeks (*k* = 1), 12 weeks (*k* = 4), and 8 weeks (*k* = 3); and IER vs. CER (*k* = 7) during 24 weeks (*k* = 1), 12 weeks (*k* = 3), and 8 weeks (*k* = 3), or IER vs. no diet (*k* = 1) during 3 weeks. Body composition was assessed with dual X-ray absorptiometry (*k* = 17), air displacement plethysmography (*k* = 3), bioimpedance (*k* = 24) or anthropometry (*k* = 22). Eighteen studies included only women and twenty-one studies tested individuals with overweight and/or obesity. Approximately 48% of the studies controlled only for dietary intake (*k* = 32) and 29 studies assessed dietary intake plus exercise. The sample sizes varied from 9 to 332 experimental participants and their mean ages ranged between 18 and 72 years.

### Quality Assessment

Supplementary Table 2 shows the detailed classification of each quality domain and the overall methodological quality of each study. Of the 66 studies identified as relevant for this systematic review, the overall methodological quality of 40 studies was rated as “moderate” and 26 were classified as “weak.” Twenty-seven studies (randomized controlled trials) were rated as strong and 13 (non-randomized controlled trials) were rated as moderate regarding the quality of study design; the remaining studies were rated as weak. All studies (*k* = 66) were classified as weak regarding selection bias (representativeness) because the samples were self-selected or composed by volunteers. With regard to the adjustment of analyses for confounders, all studies (*k* = 66) were classified as strong because there was no bias arising from lack of controlled elements. In what concerns data collection methods, all studies were rated as strong because they all used valid and reliable tools. Finally, regarding the reporting of withdrawals and dropouts, 20 studies were rated as strong, 19 studies were rated as moderate, and one study was rated as weak.

### Effects of Intermittent Fasting on Specific Outcomes

**Figure 2:**
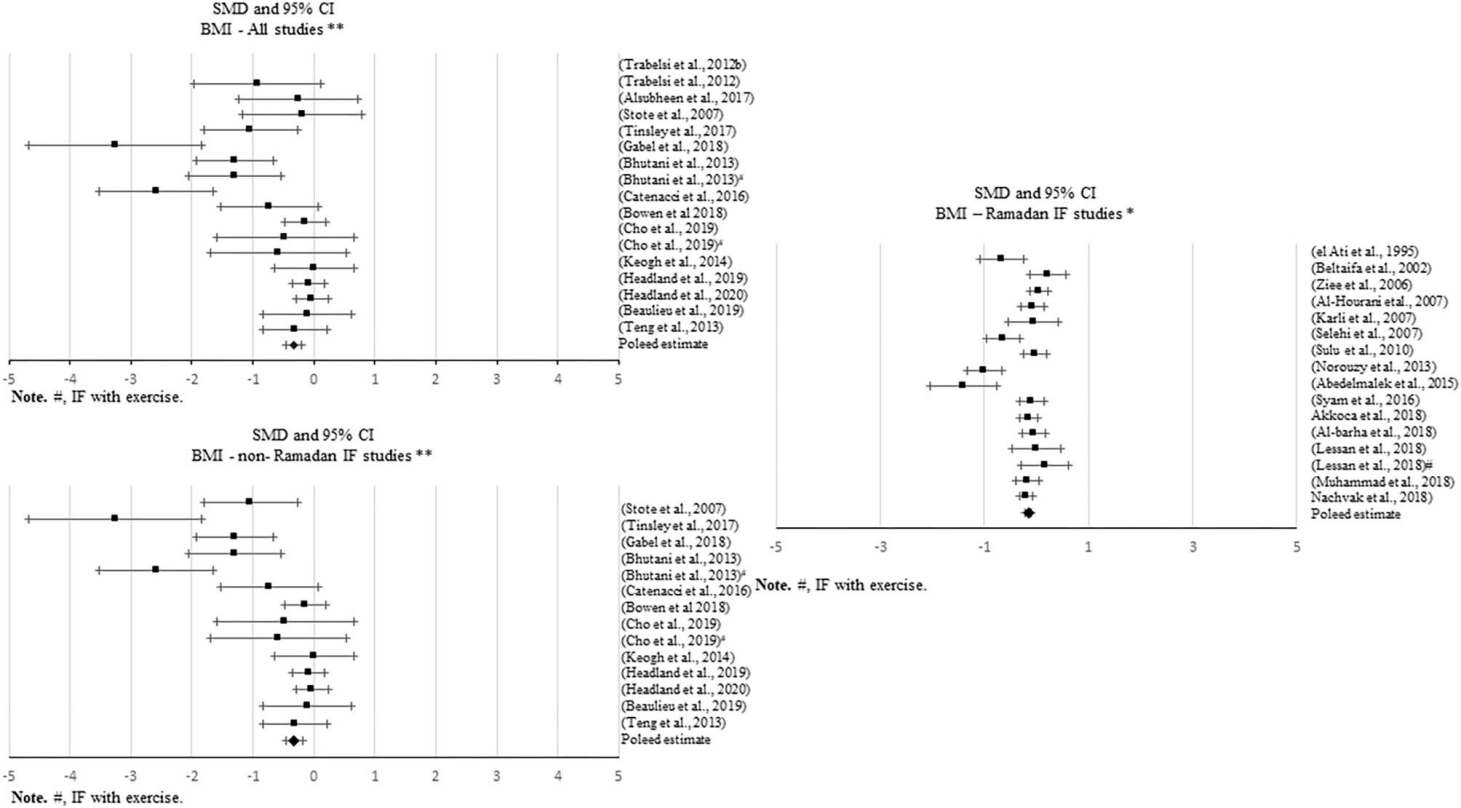
Forest plot of the effects from a random effects and fixed-effects meta-analysis shown as standardized mean difference with 95% confidence intervals on absolute body weight (BW) for all studies, only Ramadan IF and only non-Ramadan IF studies. For each study, the square represents the standardized mean difference between pre-post data intervention and control groups, with the horizontal line intersecting it as the lower and upper limits of the 95% confidence interval. Forest plot of the effects from a random effects and fixed-effects meta-analysis shown as standardized mean difference with 95% confidence intervals on absolute body weight (BW) for all studies, only Ramadan IF and only non-Ramadan IF studies. For each study, the square represents the standardized mean difference between pre- and post-intervention time points. The rhombi represents the pooled estimated standardized mean difference. *Pre-post tests, IF intervention; **Pre-post tests, IF intervention vs. control.

**Figure 3:**
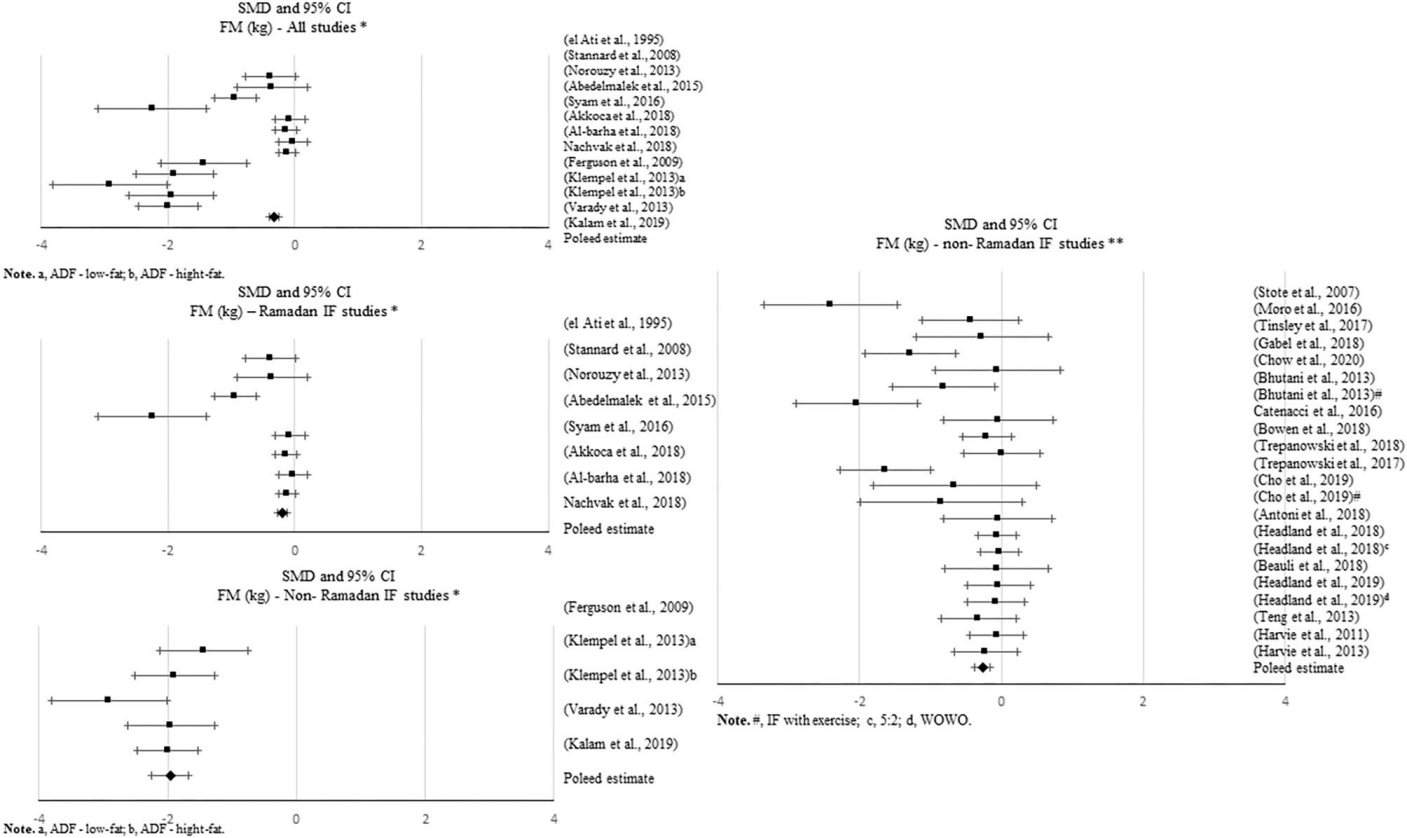
Forest plot of the effects from a random-effects meta-analysis shown as standardized mean difference with 95% confidence intervals on body mass index (BMI) for all studies and only non-Ramadan IF studies. For each study, the square represents the standardized mean difference between pre-post data intervention and control groups, with the horizontal line intersecting it as the lower and upper limits of the 95% confidence interval. Forest plot of the effects from a random-effects meta-analysis shown as standardized mean difference with 95% confidence intervals on body mass index (BMI) for only Ramadan IF studies. For each study, the square represents the standardized mean difference between pre- and post-intervention time points. The rhombi represents the pooled estimated standardized mean difference. *Pre-post tests, IF intervention; **Pre-post tests, IF intervention vs. control.

**Figure 4:**
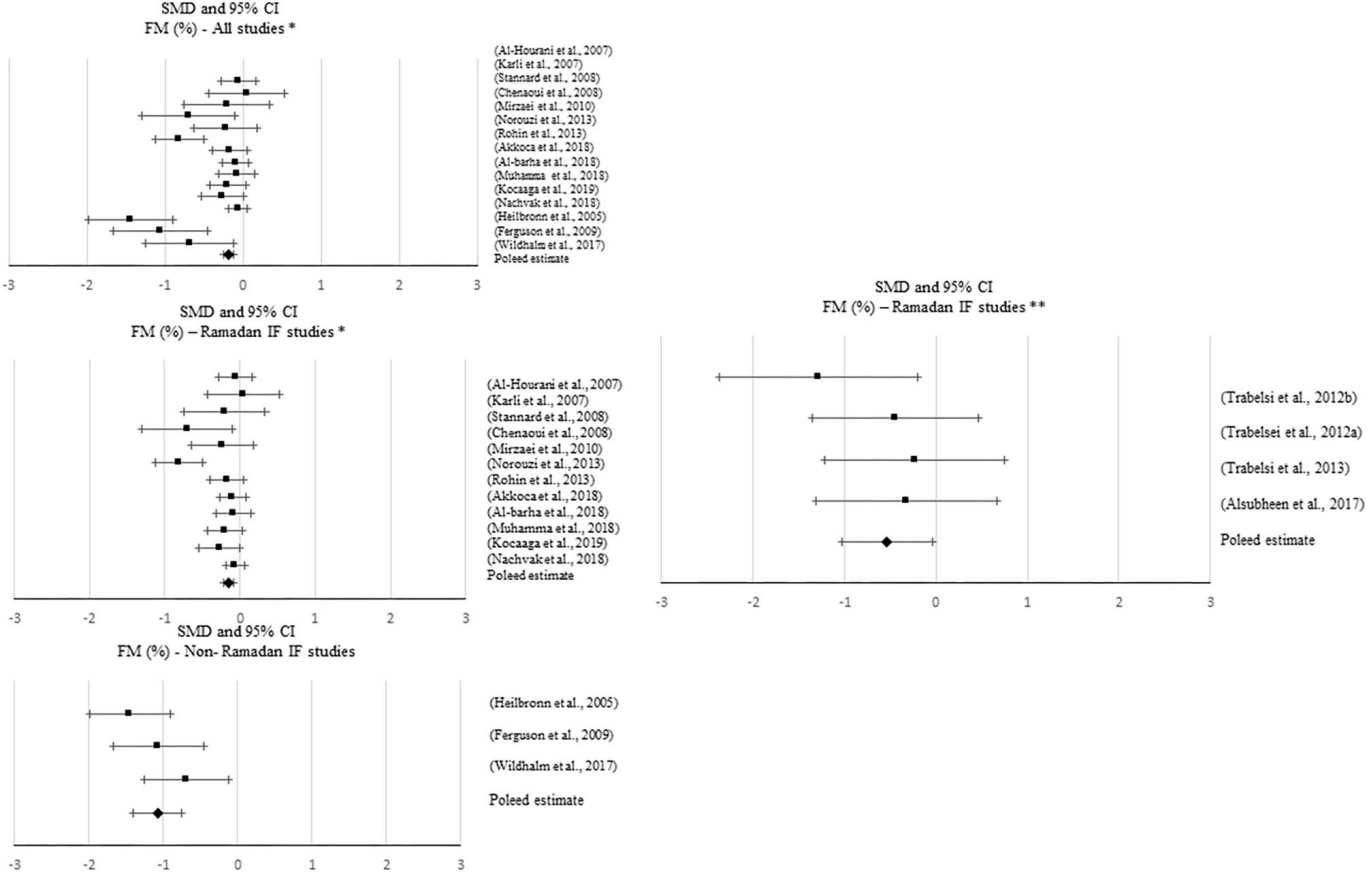
Forest plot of the effects from a random-effects meta-analysis shown as standardized mean difference with 95% confidence intervals on absolute fat mass (FM) for all studies and only non-Ramadan IF studies. For each study, the square represents the standardized mean difference between pre-post data intervention and control groups, with the horizontal line intersecting it as the lower and upper limits of the 95% confidence interval. Forest plot of the effects from a random-effects and fixed-effects, respectively, meta-analysis shown as standardized mean difference with 95% confidence intervals on absolute FM for all studies and only non-Ramadan IF studies. For each study, the square represents the standardized mean difference between between pre- and post-intervention time points. The rhombi represents the pooled estimated standardized mean difference. *Pre-post tests, IF intervention; **Pre-post tests, IF intervention vs. control.

**Figure 5:**
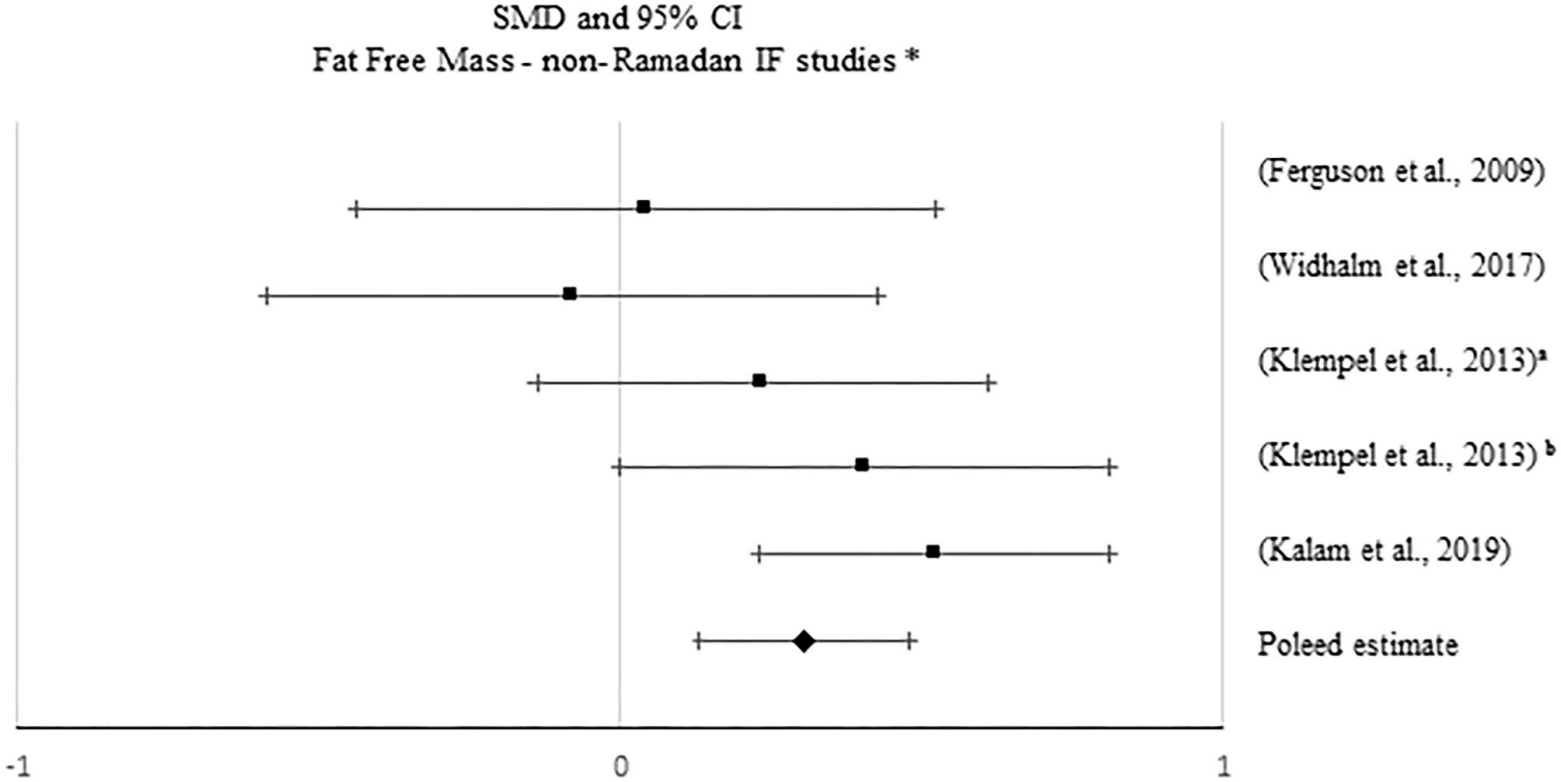
Forest plot of the effects from a random-effects and fixed-effects, respectively, meta-analysis shown as standardized mean difference with 95% confidence intervals on relative fat mass (FM) for all studies and only Ramadan IF studies. For each study, the square represents the standardized mean difference between pre-post data intervention and control groups, with the horizontal line intersecting it as the lower and upper limits of the 95% confidence interval. Forest plot of the effects from a random-effects and fixed-effects, meta-analysis shown as standardized mean difference with 95% confidence intervals on relative FM for all studies, only Ramadan IF studies and only non-Ramadan IF studies. For each study, the square represents the standardized mean difference between pre- and post-intervention time points. The rhombi represents the pooled estimated standardized mean difference. *Pre-post tests, IF intervention; **Pre-post tests, IF intervention vs. control.

**Figure 6:**
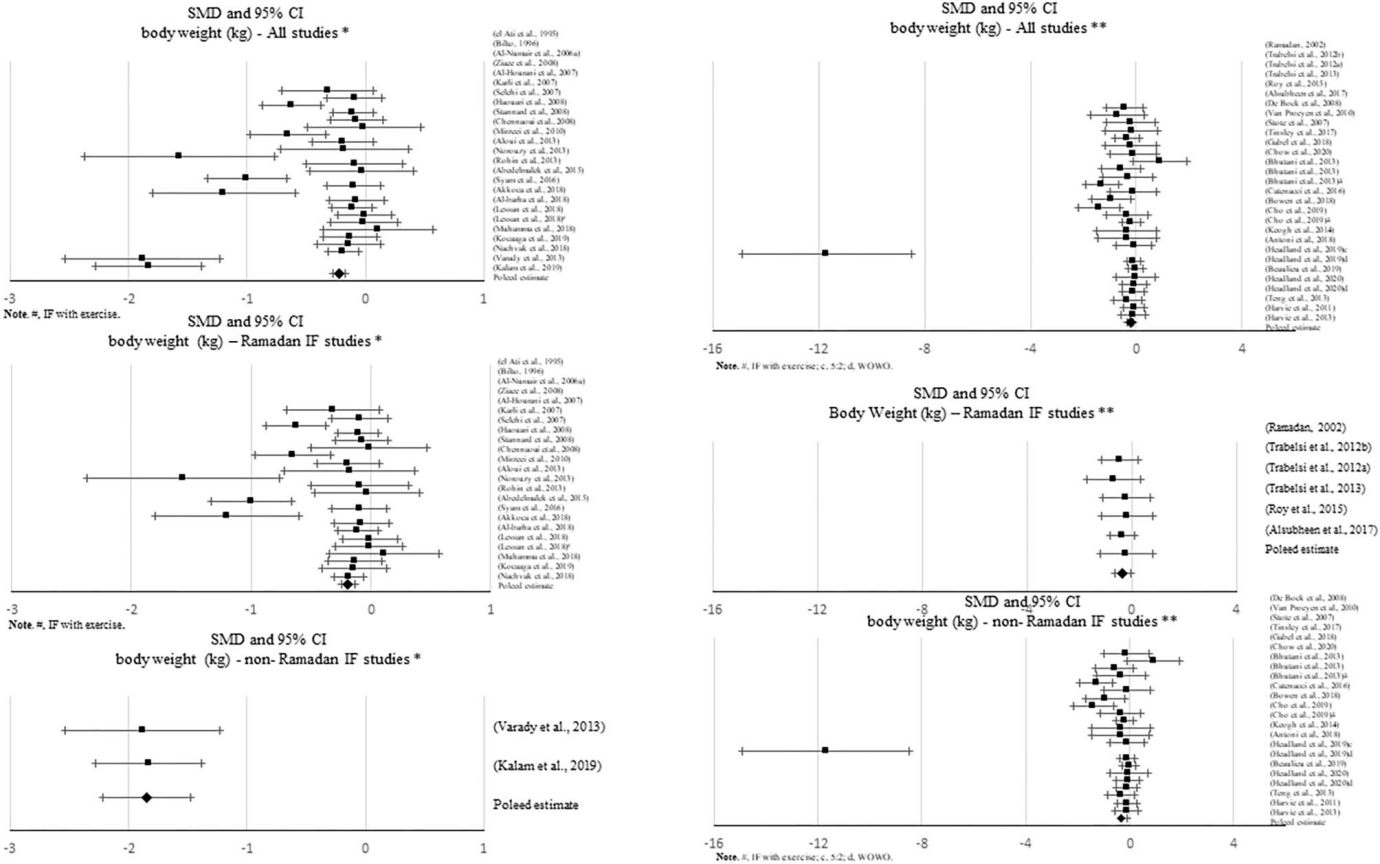
Forest plot of the effects from a fixed-effects meta-analysis shown as standardized mean difference with 95% confidence intervals on fat-free mass (FFM) for only non-Ramadan IF studies. For each study, the square represents the standardized mean difference between pre- and post-intervention time points, with the horizontal line intersecting it as the lower and upper limits of the 95% confidence interval. The rhombi represents the pooled estimated standardized mean difference. *Pre-post tests, IF intervention; **Pre-post tests, IF intervention vs. control.

A data analytic synthesis of the effects of IF on the four tested body composition outcomes (i.e., body weight, BMI, absolute and relative FM, and FFM) is shown in Supplementary Table 1. Table 1 shows the meta-analytic results for the pooled estimates of these effects, and Figures 2–6 the respective forest plots. Tables 2–4 show the moderation effect of nutritional status, exercise and caloric control, respectively, on these estimates.

**Table 1:** Meta-analytic results for the effects of intermittent fasting on body composition.

**Outcomes**	***k***	**Point estimate**	**CI lower**	**CI upper**	***P***	**Heterogeneity**		
						**Q-value**	***P***	**I^**2**^**
Body Weight (kg)^*^								
All studies	25	−0.347	−0.488	−0.205	**<0.001**	153.762	**<0.001**	84.391
Only Ramadan IF studies	23	−0.234	−0.341	−0.127	**<0.001**	77.564	**<0.001**	71.636
Only non-Ramadan IF studies	2	−1.845	−2.213	−1.477	**<0.001**	0.016	0.899	0.000
Body Weight (kg)^**^								
All studies	28	−0.334	−0.537	−0.13	**0.001**	85.219	**<0.001**	68.317
Only Ramadan IF studies	6	−0.353	−0.651	−0.054	**0.02**	0.839	0.974	0.000
Only non-Ramadan IF studies	22	−0.341	−0.584	−0.098	**0.006**	83.593	**<0.001**	74.878
**BMI (kg/m**^**2**^)^*^								
All studies								
Only Ramadan IF studies^a^	16	−0.212	−0.353	−0.072	**0.003**	69.694	**<0.001**	78.477
Only non-Ramadan IF studies								
**BMI (kg/m**^**2**^)^**^								
All studies	17	−0.655	−0.97	−0.341	**<0.001**	70.116	**<0.001**	77.181
Only Ramadan IF studies	3	−0.439	−1.017	0.138	0.136	1.229	0.541	0.000
Only non-Ramadan IF studies	14	−0.699	−1.05	−0.347	**<0.001**	68.735	**<0.001**	81.087
Fat Mass (kg)^*^								
All studies	13	−0.996	−1.351	−0.641	**<0.001**	196.692	**<0.001**	93.899
Only Ramadan IF studies	5	−0.376	−0.618	−0.135	**0.002**	47.803	**<0.001**	85.357
Only non-Ramadan IF studies	5	−1.963	−2.243	−1.682	**<0.001**	6.682	0.154	40.138
Fat Mass (kg)^**^								
All studies	24	−0.435	−0.65	−0.22	**<0.001**	78.722	**<0.001**	70.783
Only Ramadan IF studies	2	−0.286	−0.985	0.413	0.423	0.002	0.966	0.000
Only non-Ramadan IF studies	22	−0.447	−0.673	−0.221	**<0.001**	78.717	**<0.001**	73.322
Fat Mass (%)^*^								
All studies	15	−0.324	−0.477	−0.171	**<0.001**	58.248	**<0.001**	75.965
Only Ramadan IF studies^a^	12	−0.197	−0.312	−0.082	**0.001**	25.459	**0.008**	56.793
Only non-Ramadan IF studies	3	−1.073	−1.4	−0.746	**<0.001**	3.621	0.164	44.763
Fat Mass (%)^**^								
All studies	12	−0.237	−0.449	−0.025	**0.029**	6.04	0.871	0.000
Only Ramadan IF studies	4	−0.533	−1.025	−0.04	**0.034**	2.408	0.492	0.000
Only non-Ramadan IF studies	8	−0.169	−0.404	0.066	0.158	1.93	0.964	0.000
Fat Free Mass (kg)^*^								
All studies	15	−0.023	−0.193	0.146	0.788	66.465	**<0.001**	78.936
Only Ramadan IF studies	10	−0.142	−0.324	0.041	0.129	39.933	**<0.001**	77.462
Only non-Ramadan IF studies	5	0.306	0.133	0.48	**0.001**	5.869	0.209	31.843
Fat Free Mass (kg)^**^								
All studies	22	0.032	−0.122	0.186	0.684	33.663	**0.039**	37.617
Only Ramadan IF studies	5	−0.203	−0.599	0.192	0.314	0.214	0.995	0.000
Only non-Ramadan IF studies	17	0.073	−0.106	0.251	0.425	32.287	**0.009**	50.445

* *Pre-post tests, IF intervention*;

** Pre-post tests, IF intervention vs. control; *BMI, all studies (k = 16); *Body Weight (kg), all studies (k = 25); *Body Weight (kg), Ramadan IF studies (k = 23); *Body Weight (kg), non-Ramadan IF studies (k = 2); **Body Weight (kg), all studies (k = 28); ^**^Body Weight (kg), Ramadan IF studies (k = 6); **Body Weight (kg), non-Ramadan IF studies (k = 22); *BMI, Ramadan IF studies (k = 16); **BMI, all studies (k = 17); **BMI, Ramadan IF studies (k = 3); **BMI, non-Ramadan IF studies (k = 14); *Fat Mass (kg), all studies (k = 13); *Fat Mass (kg), Ramadan IF studies (k = 8); *Fat Mass (kg), non-Ramadan IF studies (k = 5); **Fat Mass (kg), all studies (k = 24); **Fat Mass (kg), Ramadan IF studies (k = 2); **Fat Mass (kg), non-Ramadan IF studies (k = 22); *Fat Mass (%), all studies (k = 15); *Fat Mass (%), Ramadan IF studies (k = 12); *Fat Mass (%), non-Ramadan IF studies (k = 3); ^**^Fat Mass (%), all studies (k = 12); **Fat Mass (%), Ramadan IF studies (k = 4); **Fat Mass (%), non-Ramadan IF studies (k = 8); *Fat Free Mass, all studies (k = 15), *Fat Free Mass, Ramadan IF studies (k = 10); *Fat Free Mass, non-Ramadan IF studies (k = 5); **Fat Free Mass, all studies (k = 22), **Fat Free Mass, Ramadan IF studies (k = 5); **Fat Free Mass, non-Ramadan IF studies (k = 17).

*CI, confidence interval; BMI, body mass index; IF, intermittent fasting*.

a *Sensitivity analysis: Results are no longer significant when excluding studies with weak methodological quality. The bold values are statistically significant values*.

**Table 2:** Subgroup analysis assessing the effect of nutritional status as a moderator of the impact of intermittent fasting on body composition.

**Moderators**	***k***	**Point estimate**	**CI lower**	**CI upper**	***P***	**Heterogeneity**		
						**Q-value**	***P***	**I^**2**^**
Body Weight (kg)^*^								
**All studies**								
Normal weight	21	−0.325	−0.488	−0.162	**<0.001**	0.535	0.465	83.885
Overweight/Obesity	4	−0.469	−0.818	−0.120	**0.008**			88.869
**Only Ramadan IF studies**								
Normal weight	19	−0.175	−0.281	−0.069	**0.001**	2.497	0.114	59.739
Overweight/Obesity	4	−0.469	−0.818	−0.12	**0.008**			88.869
Body Weight (kg)^**^								
**All studies**								
Normal weight	6	−0.384	−0.683	−0.085	**0.012**	0.083	0.773	0.000
Overweight/Obesity	17	−0.446	−0.734	−0.157	**0.002**			79.950
**Only non-Ramadan IF studies**								
Normal weight	2	−0.477	−1.053	0.099	0.104	0.009	0.924	0.000
Overweight/Obesity	17	−0.446	−0.734	−0.157	**0.002**			79.950
**BMI (kg/m**^**2**^)^*^
**Only Ramadan IF studies**								
Normal weight	11	−0.118	−0.272	0.035	0.130	1.516	0.218	66.978
Overweight/Obesity	6	−0.336	−0.646	−0.025	**0.034**			86.499
**BMI (kg/m**^**2**^)^**^
**All studies**								
Normal weight	4	−1.261	−2.275	−0.247	**0.015**	1.563	0.211	74.830
Overweight/Obesity	11	−0.574	−0.935	−0.214	**0.002**			79.285
**Only non-Ramadan IF studies**								
Normal weight	2	−2.061	−4.233	0.111	0.063	1.752	0.186	86.428
Overweight/Obesity	11	−0.574	−0.935	−0.214	**0.002**			79.285
Fat Mass (kg)^*^								
**All studies**								
Normal weight	11	−1.134	−1.605	−0.663	**<0.001**	1.696	0.193	94.131
Overweight/Obesity	2	−0.516	−1.318	0.287	0.208			95.255
**Only Ramadan IF studies**								
Normal weight	6	−0.334	−0.624	−0.045	**0.024**	0.173	0.678	80.912
Overweight/Obesity	2	−0.516	−1.318	0.287	0.208			95.255
Fat Mass (kg)^**^
**All studies**								
Normal weight	4	−0.838	−1.807	0.130	0.090	0.826	0.364	79.531
Overweight/Obesity	18	−0.377	−0.604	−0.149	**0.001**			70.702
**Only non-Ramadan IF studies**								
Normal weight	3	−1.021	−2.286	0.245	0.114	0.964	0.326	85.085
Overweight/Obesity	18	−0.377	−0.604	−0.149	**0.001**			70.702
Fat Mass (%)^*^								
**All studies**								
Normal weight	12	−0.328	−0.511	−0.146	**<0.001**	0.001	0.973	71.809
Overweight/Obesity	3	−0.336	−0.716	0.045	0.084			89.480
**Only Ramadan IF studies**								
Normal weight	9	−0.138	−0.228	−0.048	**0.003**	0.982	0.322	0.000
Overweight/Obesity	3	−0.336	−0.716	0.045	0.084			89.480
Fat Mass (%)^**^								
**All studies**								
Normal weight	4	−0.508	−0.994	−0.022	**0.040**	0.633	0.426	0.000
Overweight/Obesity	6	−1.354	−3.379	0.672	0.190			96.642
**Only non-Ramadan IF studies**								
Normal weight	1	−0.253	−1.186	0.680	0.595	0.936	0.333	0.000
Overweight/Obesity	6	−1.354	−3.379	0.672	0.190			96.642
Fat Free Mass (kg)^*^
**All studies**								
Normal weight	12	0.097	−0.022	0.216	0.109	4.142	**0.042**	28.246
Overweight/Obesity	3	−0.423	−0.91	0.064	0.088			93.218
**Only Ramadan IF studies**								
Normal weight	7	0.000	−0.116	0.116	0.999	2.745	0.098	0.000
Overweight/Obesity	3	−0.423	−0.91	0.064	0.088			93.218
Fat Free Mass (kg)^**^
**All studies**								
Normal weight	5	0.038	−0.331	0.407	0.841	2.238	0.135	0.000
Overweight/Obesity	14	−0.767	−1.754	0.221	0.128			97.762
**Only non-Ramadan IF studies**								
Normal weight	2	0.290	−0.214	0.795	0.259	3.491	0.062	3.942
Overweight/Obesity	14	−0.767	−1.754	0.221	0.128			97.762

* *Pre-post tests, IF intervention*;

** Pre-post tests, IF intervention vs. control; *BMI, Ramadan IF studies (k = 15); *Body Weight (kg), all studies (k = 25); **Body Weight (kg), all studies (k = 23); *Body Weight (kg), Ramadan IF studies (k = 23); **Body Weight (kg), non-Ramadan IF studies (k = 19); **BMI, all studies (k = 15); **BMI, non-Ramadan IF studies (k = 13); *Fat Mass (kg), all studies (k = 13); **Fat Mass (kg), all studies (k = 22); *Fat Mass (kg), Ramadan IF studies (k = 8); **Fat Mass (kg), non-Ramadan IF studies (k = 21); *Fat Mass (%), all studies (k = 15); **Fat Mass (%), all studies (k = 10); *Fat Mass (%), Ramadan IF studies (k = 12); **Fat Mass (%), non-Ramadan IF studies (k = 7); *Fat Free Mass, all studies (k = 15), **Fat Free Mass, all studies (k = 19); *Fat Free Mass, Ramadan IF studies (k = 10); **Fat Free Mass, non-Ramadan IF studies (k = 16).

*CI, confidence interval; BMI, body mass index; IF, intermittent fasting. The bold values are statistically significant values*.

**Table 3:** Subgroup analysis assessing the effect of exercise as a moderator of the impact of intermittent fasting on body composition.

**Moderators**	***K***	**Point estimate**	**CI lower**	**CI upper**	***P*-value**	**Heterogeneity**		
						**Q-value**	***P*-value**	**I-squared**
Body Weight (kg)^*^								
**All studies**								
With exercise	8	−0.127	−0.312	0.059	0.182	5.330	**0.021**	52.046
Without exercise	17	−0.430	−0.609	−0.251	**<0.001**			87.986
**Only Ramadan IF studies**								
With exercise	8	−0.127	−0.312	0.059	0.182	1.681	0.195	52.046
Without exercise	15	−0.276	−0.407	−0.146	**<0.001**			76.520
Body Weight (kg)^**^
**All studies**								
With exercise	8	−0.386	−0.788	0.016	**0.06**	0.078	0.780	48.868
Without exercise	20	−0.320	−0.557	−0.082	**0.008**			72.681
**Only Ramadan IF studies**								
With exercise	3	−0.419	−0.775	−0.064	**0.021**	0.457	0.499	0.000
Without exercise	3	−0.193	−0.744	0.357	0.491			0.000
**Only non**–**Ramadan IF studies**								
With exercise	5	−0.290	−1.048	0.469	0.454	0.016	0.900	69.912
Without exercise	17	−0.342	−0.603	−0.081	**0.010**			76.994
**BMI (kg/m**^**2**^)^*^
**Only Ramadan IF studies**								
With exercise	4	−0.037	−0.181	0.106	0.612	4.417	**0.036**	0.000
Without exercise	12	−0.280	−0.456	−0.105	**0.002**			83.232
**BMI (kg/m**^**2**^)^**^
**All studies**								
With exercise	5	−1.481	−2.581	−0.381	**0.008**	3.550	0.060	80.758
Without exercise	12	−0.396	−0.648	−0.144	**0.002**			61.208
**Only Ramadan IF studies**								
With exercise	2	−0.572	−1.286	0.142	0.117	0.381	0.537	0.000
Without exercise	1	−0.189	−1.171	0.793	0.706			0.000
**Only non-Ramadan IF studies**								
With exercise	3	−2.112	−3.634	−0.59	**0.007**	4.660	**0.031**	81.242
Without exercise	11	−0.411	−0.675	−0.147	**0.002**			64.721
FM (kg)^*^
**All studies**								
With exercise	3	−0.552	−1.305	0.200	0.150	1.793	0.181	86.618
Without exercise	10	−1.146	−1.58	−0.712	**<0.001**			94.999
**Only Ramadan IF studies**								
With exercise	2	−0.093	−0.346	0.161	0.473	3.404	0.065	13.04
Without exercise	6	−0.466	−0.772	−0.161	**0.003**			88.977
**Only non-Ramadan IF studies**								
With exercise	1	−1.434	−2.117	−0.75	**<0.001**	2.764	0.096	0.000
Without exercise	4	−2.069	−2.376	−1.762	**<0.001**			23.425
FM (kg)^**^
**All studies**								
With exercise	5	−0.779	−1.462	−0.095	**0.026**	1.279	0.258	65.227
Without exercise	19	−0.364	−0.584	−0.145	**0.001**			70.205
**Only Ramadan IF studies**								
With exercise	1	−0.271	−1.263	0.721	0.593	0.002	0.966	0.000
Without exercise	1	−0.301	−1.286	0.685	0.549			0.000
**Only non-Ramadan IF studies**								
With exercise	4	−0.897	−1.718	−0.077	**0.032**	1.487	0.223	71.000
Without exercise	18	−0.368	−0.594	−0.142	**0.001**			71.850
FM (%)^*^
**All studies**								
With exercise	9	−0.386	−0.646	−0.125	**0.004**	0.448	0.503	76.645
Without exercise	6	−0.274	−0.470	−0.079	**0.006**			78.206
**Only Ramadan IF studies**								
With exercise	7	−0.148	−0.267	−0.028	**0.016**	0.573	0.449	0.000
Without exercise	5	−0.236	−0.432	−0.04	**0.018**			79.574
**Only non-Ramadan IF studies**								
With exercise	2	−1.271	−1.674	−0.869	**<0.001**	2.749	0.097	0.000
Without exercise	1	−0.686	−1.249	−0.124	**0.017**			0.000
FM (%)^**^
**All studies**								
With exercise	5	−0.523	−0.968	−0.078	**0.021**	2.054	0.152	0.000
Without exercise	7	−0.153	−0.394	0.088	0.215			0.000
**Only Ramadan IF studies**								
With exercise	3	−0.603	−1.172	−0.034	**0.038**	0.235	0.627	7.948
Without exercise	1	−0.321	−1.307	0.665	0.524			0.000
**Only non-Ramadan IF studies**								
With exercise	2	−0.396	−1.112	0.32	0.278	0.432	0.511	0.000
Without exercise	6	−0.142	−0.391	0.107	0.263			0.000
Fat Free Mass (kg)^*^
**All studies**								
With exercise	7	0.017	−0.101	0.135	0.779	0.178	0.673	0.000
Without exercise	8	−0.054	−0.358	0.251	0.731			88.948
**Only Ramadan IF studies**								
With exercise	6	0.015	−0.106	0.137	0.806	3.282	0.070	0.000
Without exercise	4	−0.357	−0.742	0.027	0.068			89.896
**Only non-Ramadan IF studies**								
With exercise	1	0.042	−0.438	0.523	0.862	1.333	0.248	0.000
Without exercise	4	0.346	0.160	0.532	**<0.001**			33.853
Fat Free Mass (kg)^**^
**All studies**								
With exercise	6	0.015	−0.309	0.339	0.928	0.021	0.884	0.000
Without exercise	16	0.043	−0.138	0.223	0.643			48.726
**Only Ramadan IF studies**								
With exercise	4	−0.234	−0.667	0.198	0.289	0.119	0.731	0.000
Without exercise	1	−0.046	−1.026	0.934	0.927			0.000
**Only non-Ramadan IF studies**								
With exercise	2	0.337	−0.243	0.918	0.255	0.872	0.350	29.138
Without exercise	15	0.047	−0.140	0.234	0.624			52.128

* *Pre-post tests, IF intervention*;

** Pre-post tests, IF intervention vs. control; *Body Weight (kg), all studies (k = 25); **Body Weight (kg), all studies (k = 28); *Body Weight (kg), Ramadan IF studies (k = 23); **Body Weight (kg), Ramadan IF studies (k = 6); **Body Weight (kg), non-Ramadan IF studies (k = 22); **BMI, all studies (k = 17); *BMI, Ramadan IF studies (k = 16); **BMI, Ramadan IF studies (k = 3); **BMI, non-Ramadan IF studies (k = 14); *Fat Mass (kg), all studies (k = 13); **Fat Mass (kg), all studies (k = 24); *Fat Mass (kg), Ramadan IF studies (k = 8); **Fat Mass (kg), Ramadan IF studies (k = 2); *Fat Mass (kg), non-Ramadan IF studies (k = 5); **Fat Mass (kg), non-Ramadan IF studies (k = 22); *Fat Mass (%), all studies (k = 15); **Fat Mass (%), all studies (k = 12); *Fat Mass (%), Ramadan IF studies (k = 12); **Fat Mass (%), Ramadan IF studies (k = 4); *Fat Mass (%), non-Ramadan IF studies (k = 3); **Fat Mass (%), non-Ramadan IF studies (k = 2); *Fat Free Mass, all studies (k = 15), **Fat Free Mass, all studies (k = 22); *Fat Free Mass, Ramadan IF studies (k = 10); **Fat Free Mass, Ramadan IF studies (k = 5); *Fat Free Mass, non-Ramadan IF studies (k = 5); **Fat Free Mass, non-Ramadan IF studies (k = 17).

*CI, confidence interval; BMI, body mass index; IF, intermittent fasting. The bold values are statistically significant values*.

**Table 4:** Subgroup analysis assessing the effect of caloric control as a moderator of the impact of intermittent fasting on body composition.

**Moderators**	***K***	**Point estimate**	**CI lower**	**CI upper**	***P*-value**	**Heterogeneity**		
						**Q-value**	***P*-value**	**I-squared**
Body Weight (kg)^*^								
**All studies**								
With caloric control	2	−1.845	−2.213	−1.477	**<0.001**	67.857	**<0.001**	0.000
Without caloric control	23	−0.234	−0.341	−0.127	**<0.001**			71.636
Body Weight (kg)^**^
**All studies**								
With caloric control	19	−0.295	−0.547	−0.043	**0.022**	0.925	0.336	75.001
Without caloric control	9	−0.474	−0.736	−0.212	**<0.001**			6.854
**Only non-Ramadan IF studies**								
With caloric control	19	−0.295	−0.547	−0.043	**0.022**	0.630	0.427	75.001
Without caloric control	3	−0.629	−1.415	0.156	0.116			64.495
**BMI (kg/m**^**2**^)^**^
**All studies**								
With caloric control	12	−0.502	−0.814	−0.190	**0.002**	1.646	0.199	73.887
Without caloric control	5	−1.095	−1.944	−0.245	**0.012**			74.074
**Only non-Ramadan IF studies**								
With caloric control	12	−0.502	−0.814	−0.190	**0.002**	2.834	0.092	73.887
Without caloric control	5	−2.168	−4.082	−0.254	**0.026**			83.819
FM (kg)^*^
**All studies**								
With caloric control	5	−1.979	−2.351	−1.606	**<0.001**	49.961	**<0.001**	40.138
Without caloric control	8	−0.376	−0.618	−0.135	**0.002**			85.357
FM (kg)^**^
**All studies**								
With caloric control	19	−0.421	−0.655	−0.187	**<0.001**	0.092	0.762	73.632
Without caloric control	5	−0.510	−1.034	0.014	0.057			44.933
**Only non-Ramadan IF studies**								
With caloric control	10	−0.421	−0.655	−0.187	**<0.001**	0.155	0.694	73.632
Without caloric control	3	−0.593	−1.415	0.229	0.158			67.603
FM (%)^*^
**All studies**								
With caloric control	1	−1.059	−1.659	−0.459	**0.001**	6.002	**0.014**	0.000
Without caloric control	14	−0.287	−0.435	−0.14	**<0.001**			74.042
**Only non-Ramadan IF studies**								
With caloric control	1	−1.059	−1.659	−0.459	**0.001**	0.003	0.957	0.000
Without caloric control	2	−1.079	−1.47	−0.689	**<0.001**			72.359
FM (%)^**^
**All studies**								
With caloric control	6	−0.176	−0.428	0.077	0.172	0.752	0.386	0.000
Without caloric control	6	−0.382	−0.772	0.009	0.055			0.000
**Only non-Ramadan IF studies**								
With caloric control	6	−0.176	−0.428	0.077	0.172	0.020	0.888	0.000
Without caloric control	2	−0.127	−0.767	0.514	0.699			0.000
Fat Free Mass (kg)^*^
**All studies**								
With caloric control	4	0.351	0.154	0.548	**<0.001**	13.365	**<0.001**	10.401
Without caloric control	11	−0.137	−0.31	0.036	0.119			74.985
**Only non-Ramadan IF studies**								
With caloric control	4	0.358	0.173	0.542	**<0.001**	2.521	0.112	10.401
Without caloric control	1	−0.079	−0.586	0.427	0.759			0.000
Fat Free Mass (kg)^**^
**All studies**								
With caloric control	15	0.090	−0.105	0.286	0.365	1.594	0.207	56.342
Without caloric control	7	−0.144	−0.45	0.162	0.357			0.000
**Only non-Ramadan IF studies**								
With caloric control	15	0.090	−0.105	0.286	0.365	0.297	0.586	56.342
Without caloric control	2	−0.055	−0.538	0.429	0.824			0.000

* *Pre-post tests, IF intervention*;

** Pre-post tests, IF intervention vs. control; *Body Weight (kg), all studies (k = 25); **Body Weight (kg), all studies (k = 28); **Body Weight (kg), non-Ramadan IF studies (k = 22); **BMI, all studies (k = 17); **BMI, non-Ramadan IF studies (k = 14); *Fat Mass (kg), all studies (k = 13); **Fat Mass (kg), all studies (k = 24); **Fat Mass (kg), non-Ramadan IF studies (k = 22); *Fat Mass (%), all studies (k = 15); **Fat Mass (%), all studies (k = 12); *Fat Mass (%), non-Ramadan IF studies (k = 3); **Fat Mass (%), non-Ramadan IF studies (k = 8); *Fat Free Mass, all studies (k = 15), **Fat Free Mass, all studies (k = 22); *Fat Free Mass, non-Ramadan IF studies (k = 5); **Fat Free Mass, non-Ramadan IF studies (k = 17).

*CI, confidence interval; BMI, body mass index; IF, intermittent fasting. The bold values are statistically significant values*.

#### Changes in Body Weight

Sixty-four studies reported effects of IF on body weight, with ~66% of them attaining significant changes in this specific outcome. Some studies obtained significant decreases in body weight with Ramadan IF (*k* = 20) and non-Ramadan IF [TRF (*k* = 8), ADF (*k* = 8) and IER (*k* = 5)]. Approximately 23% of the studies that observed a significant decrease in body weight had an exercise intervention (*k* = 15).

When assessing changes in body weight, significant negative pooled estimates emerged, when comparing pre- post-intervention data between treatments and the control groups, including all IF studies (*k* = 28, −0.334 (95% CI [−0.537, −0.13], *p* = 0.001), only Ramadan IF studies (*k* = 6, −0.353 (95% CI [−0.651, −0.054], *p* = 0.02), and only non-Ramadan IF studies (*k* = 22, −0.341 (95% CI [−0.584, −0.098], *p* = 0.006). There was evidence of high heterogeneity between non-Ramadan IF studies (*Q* = 83.593, *p* < 0.001, *I*^2^ = 75%) and moderate heterogeneity between all studies (*Q* = 85.219, *p* < 0.001, *I*^2^ = 68%). No heterogeneity was observed between Ramadan IF studies (*Q* = 0.839, *p* = 0.974, *I*^2^ = 0%). Similar results were found when considering pre- post-intervention data only (all: *k* = 25; SMD = −0.347, 95% CI [−0.488, −0.205], *p* < 0.001 (*Q* = 153.762, *p* < 0.001, *I*^2^ = 84%); only Ramadan IF interventions: *k* = 23; SMD = −0.234, 95% CI [−0.341, −0.127], *p* < 0.001 (*Q* =77.564, *p* < 0.001, *I*^2^ = 71%); and only non-Ramadan IF interventions: *k* = 2; SMD = −1.845, 95% CI [−2.213, −1.477], *p* < 0.001 (*Q* = 0.016, *p* = 0.899, *I*^2^ = 0%). Similar results were observed when excluding studies with weak methodological quality.

There was evidence of publication bias based on the visual inspections of the funnel plots for body weight, when comparing pre-post data between treatments and controls including all studies and non-Ramadan IF studies only, and when considering pre-post intervention data only for all studies, but due to the moderate-to-high heterogeneity observed, there was not enough power to differentiate chance from real asymmetry.

Subgroup analyses showed a moderation effect of exercise on the impact of IF on body weight when considering pre- post-intervention data for all studies (*Q* = 5.330, *p* = 0.021): a small negative effect was found when exercise was not included (*k* = 17; SMD = −0.430, 95% CI [−0.609, −0.251], *p* < 0.001). Additionally, significant differences were found between interventions with and without caloric control when considering pre- post-intervention data for all studies (*Q* = 67.857, *p* < 0.001): significantly higher negative effects emerged when calories were taken into account: *k* = 2; SMD = −1.845, 95% CI [−2.213, −1.477], *p* < 0.001 vs. *k* = 23; SMD = −0.234, 95% CI [−0.341, −0.127], *p* < 0.001).

#### Changes in Body Mass Index

Thirty-six studies presented data for the effect of IF on BMI, with ~69% of the studies showing significant changes. Most of these studies reported positive results, i.e., a significant decrease in BMI with Ramadan IF (*k* = 12) and non-Ramadan IF [TRF (*k* = 4), ADF (*k* = 5), and IER (*k* = 3)]. Only one study reported an increase in BMI with Ramadan IF. Approximately 16% of the studies showing a significant decrease in BMI had an exercise intervention (*k* = 6).

Significant decreases in BMI were found when quantitatively comparing pre- post-intervention data between IF treatments and the control groups, including all studies (*k* = 17; SMD = −0.655, 95% CI [−0.97, −0.341], *p* < 0.001) and non-Ramadan IF studies only (*k* = 14; SMD = −0.699, 95% CI [−1.05, −0.347], *p* < 0.001). There was evidence of high heterogeneity between all studies (Q = 70.116, *p* < 0.001; I^2^ = 77%) and non-Ramadan studies only (Q = 68.735, *p* < 0.001; I^2^ = 81%). Non-significant results emerged when considering Ramadan IF studies only (*p* > 0.05). Although this pooled estimate is significant when meta-analyzing pre- post-intervention data only (*k* = 16; SMD = −0.212, 95% CI [−0.353, −0.072], *p* = 0.003; Q = 69.694, *p* < 0.001; I^2^ = 78%), sensitivity analysis showed that when excluding the studies with weak methodological quality, it became non-significant (*p* > 0.05).

There was evidence of publication bias based on the visual inspection of the funnel plots, but due to the large heterogeneity observed including all studies and non-Ramadan IF studies only, there was not enough power to differentiate chance from real asymmetry.

Subgroup analyses showed that exercise moderated the effect of IF on BMI when comparing pre- post-intervention data between non-Ramadan IF treatments and the control groups (*Q* = 4.660, *p* = 0.031): a higher negative effect was found when individuals exercised (*k* = 3; SMD = −2.112, 95% CI [−3.634, −0.59], *p* = 0.007 vs. *k* = 11; SMD = −0.411, 95% CI [−0.675, −0.147], *p* = 0.002).

#### Changes in Fat Mass

A total of 49 studies reported on the effect of an IF dietary intervention on FM. Six studies reported satisfactory results as a significant decrease in absolute FM with Ramadan IF and 18 studies with non-Ramadan IF [five studies with TRF, five studies with IER, and eight studies with ADF]. Approximately 13% of the studies that observed a significant decrease in FM had an exercise intervention (*k* = 6).

Meta-analytic results showed a negative, small but significant effect of IF on absolute FM when comparing pre- post-intervention data between IF treatments and the control groups, including all studies (*k* = 24; SMD = −0.435, 95% CI [−0.65, −0.22], *p* < 0.001; Q = 78.722, *p* < 0.001; I^2^ = 70%) and non-Ramadan IF studies only (*k* = 22; SMD = −0.447, 95% CI [−0.673, −0.221], *p* < 0.001; Q = 78.717, *p* < 0.001; I^2^ = 73%). When considering pre- post-intervention data, significant pooled effects were observed with all IF interventions (*k* = 13; SMD = −0.996, 95% CI [−1.351, −0.641], *p* < 0.001; Q = 196.692, *p* < 0.001; I^2^ = 94%), Ramadan IF interventions only (*k* = 8; SMD = −0.376, 95% CI [−0.618, −0.135], *p* = 0.002; Q = 47.803, *p* < 0.001; I^2^ = 85%) and non-Ramadan IF interventions only (*k* = 5; SMD = −1.963, 95% CI [−2.243, −1.682], *p* < 0.001; Q = 6.682, *p* = 0.154; I^2^ = 40%). Similar results were found after conducting sensitivity analyses (i.e., removing studies with weak methodological quality).

Results of the meta-analyses for relative FM showed a negative, moderate effect when comparing pre- post-intervention data between Ramadan IF treatments and the control groups (*k* = 4; SMD = −0.533, 95% CI [−1.025, −0.04], *p* = 0.034; Q = 2.408, *p* = 0.492; I^2^ = 0%) and a negative, but small effect when comparing pre- post-intervention data between all IF treatments and the control groups (*k* = 12; SMD = −0.237, 95% CI [−0.449, −0.025], *p* = 0.029; Q = 6.04, *p* = 0.871; I^2^ = 0%). Again, when considering pre- post-intervention data only, significant pooled estimates were observed with all IF interventions (*p* ≤ 0.001).

Sensitivity analyses showed that, when excluding the studies with poor quality, the effect of Ramadan IF interventions on relative FM became non-significant (*p* > 0.05).

Regarding publication bias, visual inspections of the funnel plots for absolute FM showed the presence of asymmetry between all studies and between non-Ramadan IF studies only, when comparing pre- post-intervention data between treatments and controls, and between all studies when considering pre- post-intervention data only. Visual inspections of the funnel plots for relative FM also demonstrated asymmetry between all studies, when considering pre- post-intervention data only. However, due to the moderate-to-high heterogeneity observed, there was not enough power to differentiate chance from real asymmetry.

Subgroup analyses showed that, when considering pre- post-intervention data for all studies, there were significant differences in the effect of IF on absolute and relative FM between studies with and without caloric control (absolute FM: *Q* = 49.961, *p* < 0.001; FM (%): *Q* = 6.002, *p* = 0.014): significantly higher negative effects were observed when calories were taken into account (absolute FM: *k* = 5; SMD = −1.979, 95% CI [−2.351, −1.606], *p* < 0.001 vs. *k* = 8; SMD = −0.376, 95% CI [−0.618, −0.135], *p* = 0.002; relative FM: *k* = 1; SMD = −1.059, 95% CI [−1.659, −0.459], *p* = 0.001 vs. *k* = 14; SMD = −0.287, 95% CI [−0.435, −0.14], *p* < 0.001).

#### Changes in Fat-Free Mass

Approximately 62% of the studies reported effects of IF on FFM (*k* = 41). Only one study (TRF protocol) showed a significant increase in FFM. Fourteen studies reported significant decreases in this specific outcome with Ramadan IF (*k* = 3) and non-Ramadan IF [TRF (*k* = 2), ADF (*k* = 5), and IER (*k* = 4)]. Only 2 and 7% of the studies showing, respectively, significant increases and decreases in FFM, included exercise.

No significant pooled estimates emerged when comparing pre- post-intervention data between the IF treatments and the control groups (*p* > 0.05). However, when meta-analyzing pre- post-intervention data, a significant increase in FFM was found with non-Ramadan IF interventions (*k* = 5; SMD = 0.306, 95% CI [0.133, 0.48], *p* = 0.001; *Q* = 5.869, *p* = 0.209; I^2^ = 32%). Sensitivity analysis revealed no significant changes.

Regarding publication bias, visual inspections of the funnel plots did not show the presence of asymmetry, which was confirmed with Egger's tests (*p* > 0.05).

Subgroups analyses revealed significant differences in the effect of IF on FFM between individuals with normal weight and overweight, when considering pre- post-intervention data for all studies (*Q* = 4.142, *p* = 0.042), although changes in each subgroup were not significant (*p* > 0.05). Significant differences in the effect of IF on FFM between studies with and without caloric control were also found, when considering pre- post-intervention data for all studies (*Q* = 13.365, *p* < 0.001): a small positive effect was found when caloric intake was taken into account (*k* = 4; SMD = 0.351, 95% CI [0.154, 0.548], *p* < 0.001).

## Discussion

This study sought to summarize the effects of different IF approaches on body composition-related outcomes. To our knowledge, this is the first systematic review and meta-analysis presenting comprehensive results on changes in specific parameters of body composition resulting from Ramadan vs. non-Ramadan IF, which is of considerable relevance given the recent popularity of these approaches among researchers and clinical nutritionists/sports physiologists.

Our findings showed that, when comparing pre- with post-intervention data between treatment and control groups, Ramadan IF elicits significant reductions in some body composition parameters, namely in relative FM and body weight. Similar results were also found when considering pre- to post-intervention (within-subjects design); specifically, data indicated that Ramadan IF leads to significant reductions in BMI, FM (relative and absolute), and body weight. Our findings also suggest that, with Ramadan IF, weight loss seems to be more pronounced in persons exhibiting higher BMI values, although there were no significant differences between groups (normal weight vs. overweight/obesity). These findings are in line with those of past research (30). A greater loss of body fluids in individuals with overweight/obesity during Ramadan IF offers a likely explanation for such relationships (52).

In the general population, weight loss subsequent to Ramadan IF is largely caused by the unconscious restriction of food intake. Under these circumstances, the reduction in meal frequency is causally linked with the prolonged daily periods of standing and praying (19, 24, 26, 31, 32, 36). In addition, there is a metabolic shift toward the predominant use of fatty acids as fuel for adenosine triphosphate (ATP) synthesis during Ramadan IF and this lowers body fat (25). The optimization of energy reserves during this type of IF may also serve as a possible explanation for this phenomenon. According to this concept, while being effective in preserving protein pools via a reduction in basal metabolism, Ramadan IF also leads to a decreased secretion of anabolic hormones (e.g., insulin) and an increased secretion of catabolic hormones (i.e., adrenaline and glucagon) (53). Interestingly, it has been contended that weight loss resulting from Ramadan IF is also accompanied by reductions in FFM (>35% in normoponderal people and ~20–30% in those with overweight/obesity) (54). Our meta-analytic findings do not support this contention, revealing no significant effects of Ramadan IF on FFM. From a physiological standpoint, this is a relevant finding because FFM is well-known to play an important role on functional capacity, resting energy expenditure and blood glucose homeostasis (55). However, despite representing an important opportunity for some people to lose body weight and FM, the adaptations inherent to Ramadan IF are typically transient and largely reversible within a small amount of time (26). Thus, the prescription of maintenance strategies at termination of Ramadan is of extreme value for the long-term preservation of healthy body composition.

Caloric restriction and different forms of fasting are well-known to exert a powerful physiological impact on humans, namely on health status and body composition (5). According to our analyses, more than half of the studies (pre- post-intervention within-subjects design) examining the effects of non-Ramadan IF on FFM revealed small, but significant improvements in FFM. TRF was shown to be particularly effective in inducing slight increases in FFM [e.g., (17, 56)]. In fact, it has been argued that, when combined with resistance training, TRF has an adjunctive role in preserving or delaying possible FFM losses (3, 57). Moreover, fasting triggers numerous endocrine responses that may well contribute for heightened metabolic rate and preserved FFM (e.g., increased serum levels of noradrenaline and growth hormone, respectively) (58). In addition, exercising while fasting increases the expression of sirtuin 1 (SIRT1) as well as the phosphorylation of adenosine monophosphate-activated protein kinase (AMPK), both of which have numerous effects on gene expression (i.e., regulation of mitochondrial biogenesis) (59). Yet, it has been disputed whether the interaction between TRF and gains in FFM might be mediated by the amount of dietary protein intake, together with the final energy balance achieved on a daily basis (3, 60). This is partially supported by our moderation analyses because, when calorie consumption was taken into consideration, a significant effect of IF on FFM emerged. In addition, when comparing pre- post-intervention data between treatment and control groups, the beneficial effects of IF on decreasing BMI were more pronounced after accounting for exercise training. Non-Ramadan IF also led to significant reductions in body weight and absolute FM when comparing pre- post-intervention data between treatment and control groups, as well as when comparing pre- post-intervention on within-subjects designs. In fact, studies specifically designed to test the effects of IF on body composition have consistently shown that different approaches of IF (i.e., ADF, IER, and TRF) are well-suited for managing successful losses in body weight and FM (2, 10, 13, 61). One possible explanation is that 12 h of fasting generate a lipolytic-prone state that is compatible with decreased fat storage and enhanced fatty acid mobilization, together with ketogenesis (62). Moreover, in the short term, non-Ramadan IF (i.e., ADF protocol 5:2) appears to be superior (due to higher compliance) than CER for reducing FM (61).

According to the present beliefs, Ramadan IF is more effective for weight loss than non-Ramadan IF due to a greater loss of body water resulting from fluid deprivation (53). Although the opposite was found when considering our meta-analytical results involving pre- post-intervention analyses (within-subjects designs) (i.e., higher effects derived from non-Ramadan IF), similar results were observed for Ramadan vs. non-Ramadan IF interventions when comparing treatments and controls. However, there are clear differences in what concerns to absolute FM, with non-Ramadan IF showing greater improvements in this specific parameter, therefore being unequivocally linked to improved body composition.

This study has some limitations. First, our findings should be interpreted with caution because analyses were carried out on different populations. Second, since we did not include individuals from all ages, ethnicities, religions, geographical areas and educational backgrounds, our findings cannot be extrapolated to the general population. Third, the degree of calorie restriction varied between studies and the non-fasting control groups also ranged from “normal diet” to CER. Fourth, the nutritional profile of foods consumed during the *ad libitum* feeding period should be further monitored. Fifth, the analyses of body composition were made with different techniques and, due to the limited number of studies per technique, it was not possible to conduct moderation analyses. Bioimpedance, a technique that assumes individual euhydration, was used in many studies. Since Ramadan IF is typically accompanied by dehydration, in this specific context, the assessments of body composition using this method may be affected. The preferable use of gold standard techniques, such as the four-component model or even the single use of dual x-ray absorptiometry (only 16 studies here included used this method) in future research is fundamental. This approach would greatly reduce the possibility of measurement inaccuracies. Finally, it would have been important to discriminate the moderating effects of different types of exercise (e.g., endurance vs. resistance training). However, the low number of studies on each specific type of exercise precluded us from conducting separate moderation analyses. Instead, all exercise studies were compiled and used as a single moderator.

In summary, our meta-analytic findings revealed a negative significant pooled estimate effect of both Ramadan and non-Ramadan IF on FM and body weight. However, we found that decreases in FM are more pronounced with non-Ramadan IF and this type of IF, combined with exercise training, leads to higher decreases in BMI. Our data also provide preliminary evidence that non-Ramadan IF may be well-suited for eliciting small increases in FFM, particularly under circumstances involving the simultaneous control of caloric intake. Thus, although Ramadan IF certainly implicates some beneficial adaptations in weight management, non-Ramadan IF appears to be more effective in improving overall body composition (particularly, if combined with exercise training and controlled calorie intake), despite the fact it does not imply dehydration.

## Data Availability Statement

The original contributions presented in the study are included in the article/Supplementary Material, further inquiries can be directed to the corresponding author/s.

## Author Contributions

JC, IS, GM, PP-C, and AS designed the research and wrote the paper. JC, IS, and GM conducted the research, analyzed the data, and performed the statistical analysis. All authors had equal responsibility for the final content of the paper, read, and agreed to the published version of the manuscript.

## Conflict of Interest

The authors declare that the research was conducted in the absence of any commercial or financial relationships that could be construed as a potential conflict of interest.

## Abbreviations

IF: intermittent fasting
Ramadan IF: Ramadan intermittent fasting
TRF: time-restricted feeding
ADF: alternate-day fasting
IER: intermittent energy restriction
CER: continuous energy restriction
BMI: body mass index
FM: fat mass
FFM: fat-free mass
ATP: adenosine triphosphate
SMD: standardized mean difference
PRISMA: preferred reporting items for systematic reviews and meta-analysis
EPHPP: effective public health practice project
CMA: comprehensive meta-analysis
CI: confidence interval.
