# Supplementary material for: Effects of Ramadan and Non-ramadan Intermittent Fasting on Body Composition: A Systematic Review and Meta-Analysis
Source: Front Nutr. 2021 Jan 26;7:625240. doi: 10.3389/fnut.2020.625240 (PMC7870696; doi:10.3389/fnut.2020.625240)
Supplement: Supplementary file 1 [file Table_1.pdf]

Supplementary table. 1 Characteristics of the studies included in Systematic Review.

Body Composition Indicators

**Study**	**Participants**	**Age**	**Study Design**	**Intervention Protocol**	**Intervention Methods**	**Control Protocol**	**Control Methods**	**Duration**	**Body Composition assessment techniques**	**Body weight (kg)**	**BMI (kg/m2)**	**Fat mass (kg)**	**Fat mass (%)**	**Fat free mass (kg)**
(el Ati et al., 1995)	16 women	25-39 yrs	Observational design	Ramadan Intermittent Fasting.	Abstain from food and drink between dawn and sunset during Ramadan.	Before and after Ramadan.	Consuming *ad libitum* every day.	2 d before Ramadan.<br>2nd d of Ramadan.<br>End of Ramadan.<br>1 month after Ramadan	Anthropometry	No significant changes.	No significant changes	No significant changes	d.s	No significant changes
(Bilto, 1998)	43 healthy adults (34 male and 9 females)	20-48 yrs	Observational design	Ramadan Intermittent Fasting.	Abstain from food and drink between dawn and sunset during Ramadan.	Before and after Ramadan.	Consuming ad libitum every day.	1 wk before Ramadan.<br>2nd wk of Ramadan.<br>End of Ramadan	Anthropometry	Significant ↓in BW (1.2kg) post-Ramadan.	d.s	d.s	d.s	d.s
(Ramadan et al., 1999)	6 physically active men (A).<br>7 sedentary men (S).	Group A, 35.5 ± 1.6 yrs<br>Group S, 37.6	Observational design	Ramadan Intermittent Fasting.	Abstain from food and drink between dawn and sunset during Ramadan.	Before and after Ramadan.	Consuming ad libitum every day.	1 wk before Ramadan.<br>2 wks after the	Anthropometry	↓ non-significantly in both groups (lost less than 1 kg).	d.s	d.s	↓ non-significantly in both groups.	d.s

		± 2.3 yrs						start of Ramadan.<br><br>4th wk of Ramadan.						
(Beltaifa et al., 2002)	20 healthy adults (12 males and 8 females)	43 ± 14 yrs	Observational design	Ramadan Intermittent Fasting.	Abstain from food and drink between dawn and sunset during Ramadan.	Before and after Ramadan.	Consuming ad libitum every day.	Pre-Ramadan.<br><br>During Ramadan<br><br>Post-Ramadan.	Anthropometry	d.s	No significant changes for BMI.	d.s	d.s	d.s
(Ramadan, 2002)	16 sedentary adults Kuwaiti males	35.0 ± 1.9 yrs	Quasi-experimental design	Ramadan Intermittent Fasting.	Abstain from food and drink between dawn and sunset during Ramadan.	Pre- non-Ramadan.<br><br>Post- non-Ramadan.	Maintain regular food habits.	Pre-Ramadan.<br><br>Post-Ramadan.	Anthropometry	No significant changes in EG and CG.	d.s	d.s	No significant changes in EG and CG.	No significant changes in EG and CG.
(Heilbronn et al., 2005)	16 nonobese subjects (8 men and 8 women)	Men 34 ± 3 yrs Women 30 ± 1 yrs	RCT	ADF	Fast during 24h on alternate days.	ND	Maintain regular food habits (to maintain their BW, they were informed to double their usual food intake).	3 wks	DXA	d.s	d.s	d.s	Significant ↓ in FM (4%).	Significant ↓ in FFM.
(Al-Numair, 2006a)	45 healthy men	30-45 yrs	Observational design	Ramadan Intermittent Fasting.	Abstain from food and drink between dawn and sunset during Ramadan.	Before and after Ramadan.	Consuming ad libitum every day.	1 wk before Ramadan.<br><br>4th wk of Ramadan.	Anthropometry	Significant ↓in BW (2.3kg) post-Ramadan.	d.s	d.s	d.s	d.s
(Ziaee et al., 2006)	80 healthy adults (41 males and 39 females)	18-39 yrs	Observational design	Ramadan Intermittent Fasting.	Abstain from food	Before and after Ramadan.	Consuming ad libitum every day.	3 d before	Anthropometry	Significant ↓in BW (2.7kg)	↓in BMI post-Ramadan	d.s	d.s	d.s

					and drink between dawn and sunset during Ramadan.			Ramadan.<br><br>4th wk of Ramadan.		post-Ramadan.	(bur not significant).			
(Al-Hourani and Atoum, 2007)	57 female students	18-29 yrs	Observational design	Ramadan Intermittent Fasting.	Abstain from food and drink between dawn and sunset during Ramadan.	Before and after Ramadan.	Consuming ad libitum every day.	1 wk before Ramadan.<br><br>1 wk after the start of Ramadan.<br><br>2 wks after the start of Ramadan.<br><br>4th wk of Ramadan.	Bioimpedance	Significant ↓in BW (0.6kg) post-Ramadan.	Significant ↓in BMI (0.2kg) post-Ramadan.	d.s	No significant changes.	No significant changes.
(Karli et al., 2007)	10 male elite power athletes	22.30 ± 1.25 yrs	Observational design	Ramadan Intermittent Fasting.	Abstain from food and drink between dawn and sunset during Ramadan.	Before and after Ramadan.	Consuming ad libitum every day.	3 d before Ramadan.<br><br>End of Ramadan.<br><br>3 d after Ramadan.	Bioimpedance	No significant changes at the end of Ramadan.	No significant changes at the end of Ramadan.	d.s	No significant changes at the end of Ramadan.	No significant changes at the end of Ramadan.
(Salehi and Neghab, 2007)	28 overweight males	20-26 yrs	Non-Randomized control trial	Ramadan Intermittent Fasting.	Abstain from food and drink between dawn and sunset during Ramadan.	Before and after Ramadan.	Consuming ad libitum every day (free diet).	1 d before Ramadan.	Anthropometry	Significant ↓in BW (5.07kg) 1 d after Ramadan.	Significant ↓in BMI (1.56kg) 1 d after Ramadan.	d.s	d.s	d.s

					Diet, 2000Kcal.			1 d after Ramadan.						
(Stote et al., 2007)	15 healthy, normal-weight adults.	40-50 yrs	RCT	TRF	20-h of fasting and 4-h of feeding per day.<br><br>To maintain constant body weight during the study, energy intake was adjusted in 200 kcal increments.	Control diet	3 meals/d (breakfast, lunch, and dinner).<br><br>The subjects were fed at an energy intake that would maintain BW.	8 wks	Bioimpedance	Significant ↓in BW (1.4 kg) at the end of 8 wks in TRF group when compared to CG.	Significant ↓in BMI (0.4kg) at the end of 8 wks in TRF group when compared to CG.	Significant ↓in FM (2.1 kg) at the end of 8 wks in TRF group when compared to CG.	d.s	No significant ↑ in FFM at the end of 8 wks (p = 0.06) in TRF group when compared to CG.
(Haouari et al., 2008)	36 male student.	24 ± 1.6 yrs	Observational design	Ramadan Intermittent Fasting.	Abstain from food and drink between dawn and sunset during Ramadan.	Before and after Ramadan.	Consuming ad libitum every day.	1 wk before Ramadan.<br><br>21st d of Ramadan.	Bioimpedance	No significant changes at 21st d of Ramadan.	d.s	d.s	d.s	d.s
(De Bock et al., 2008)	20 healthy, physically active men.	21.2 ± 0.4 yrs	Non-Randomized control trial	FAST (TRF)	11-h overnight fast.	CHO	CHO intake before and during exercise (2865 ± 151 Kcal/d).	6 wks	Anthropometry	No significant changes within and between groups.	d.s	d.s	d.s	d.s
(Stannard and Thompson, 2008)	8 physically active, healthy Muslim men	21-41 yrs	Observational design	Ramadan Intermittent Fasting.	Abstain from food and drink between dawn and sunset during Ramadan.	Before and after Ramadan.	Consuming ad libitum every day.	1 wk before Ramadan.<br><br>1 wks after the start of Ramadan.<br><br>4th wk of Ramadan.	Anthropometry	Significant ↓in BW (1.27kg) at the end of Ramadan.	d.s	Significant ↓in FM (0.7kg) at the end of Ramadan.	No significant changes at the end of Ramadan.	No significant changes at the end of Ramadan.

(Chennaoui et al., 2009)	8 middle-distance athletes.	25.0 ± 1.3 yrs	Observational design	Ramadan Intermittent Fasting.	Abstain from food and drink between dawn and sunset during Ramadan.	Before and after Ramadan.	Consuming ad libitum every day.	5 d before Ramadan.<br><br>7th d of Ramadan.<br><br>21st d of Ramadan.<br><br>End of Ramadan.	Anthropometry	No significant changes at the end of Ramadan.	d.s	d.s	No significant changes at the end of Ramadan.	d.s
(Ferguson et al., 2009)	10 trained, healthy competitive cyclists.	36 ± 1 yrs	Non-Randomized control trial	IER	Cycling in an overnight fast state after 3 wks of 40% CR.	CG	Participants were their own controls.	3 wks	Air displacement plethysmography	Significant ↓in BW (1.7kg) after the CR period.	d.s	d.s	Significant ↓in FM (2.1%) after the CR period.	No significant ↑ in FFM after the CR period.
(SÜLÜ et al., 2010)	45 healthy volunteers (23 males; 22 females)	21-51 yrs	Observational design	Ramadan Intermittent Fasting.	Abstain from food and drink between dawn and sunset during Ramadan.	Before and after Ramadan.	Consuming ad libitum every day.	Before Ramadan.<br><br>End of Ramadan.	Anthropometry	↓ non-significantly at the end of Ramadan.	↓ non-significantly at the end of Ramadan.	d.s	d.s	d.s
(Van Proeyen et al., 2010)	27 healthy male volunteers (FAST, n = 10; CHO, n = 10; CG, n = 7)	18-25yrs	RCT	FAST (TRF)<br><br>CHO	Training 4 d/wk in the fasted state (2911 Kcal/d).<br><br>Ingested CHO before and during the training sessions (3012 Kcal/d).	CG	Hyper-caloric fat-rich diet (3081 Kcal/d) and did not training.	6 wks	Anthropometry	Significant ↑ in BW (3kg) in CG.<br><br>Significant ↓in BW (1.4kg) in CHO group.<br><br>No significant changes in FAST group post-test.	d.s	d.s	d.s	d.s

(Harvie et al., 2011)	107 women (IER, n = 53; CER, n = 54)	30-45 yrs	RCT	IER	25% restriction delivered as a VLCD for 2 days/week with no restriction on the other 5 d/wk	CER	25% restriction below estimated requirements 7 d/wk	24 wks	Bioimpedance	No significant changes between groups.	d.s	No significant changes between groups.	No significant changes between groups.	No significant changes between groups.
(Hajek et al., 2012)	202 participants (n = 115, Before and after Ramadan; n = 87 before and 1 month later of Ramadan)	34.3 ± 11.3 yrs	Quasi-experimental design	Ramadan Intermittent Fasting.	Abstain from food and drink between dawn and sunset during Ramadan.	Before and after Ramadan (1 month later).	Consuming ad libitum every day.	6 d before Ramadan.<br><br>End of Ramadan.	Anthropometry	Significant ↓in BW (0.84kg) at the end of Ramadan.	d.s	d.s	d.s	d.s
(Mirzaei et al., 2012)	14 male collegiate wrestlers	20.1 ± 2.5	Observational design	Ramadan Intermittent Fasting.	Abstain from food and drink between dawn and sunset during Ramadan.	Before and after Ramadan.	Consuming ad libitum every day.	1 wk before Ramadan.<br><br>End of Ramadan.	Bioimpedance	Significant ↓in BW (1.65kg) at the end of Ramadan.	d.s	d.s	Significant ↓in FM (1.13%) at the end of Ramadan.	Significant ↓in FFM (0.66kg) at the end of Ramadan.
(Trabelsi et al., 2012b)	16 male bodybuilders (n = 9, fasters; n = 7, Non-fasters)	Fasters, 24 ± 3 yrs.<br><br>Non-fasters 26 ± 3 yrs.	Non-Randomized control trial	Ramadan Intermittent Fasting.	Abstain from food and drink between dawn and sunset during Ramadan.	Before Ramadan.<br><br>End of Ramadan.	Consuming ad libitum every day.	Before Ramadan.<br><br>End of Ramadan.	Anthropometry	Significant ↑ in BW (1.9kg) at the end of Ramadan in Non-fasters group.	Significant ↑ in BMI (0.6kg) at the end of Ramadan in Non-fasters group.	No significant changes in both groups.	No significant changes in both groups.	No significant changes in both groups.
(Trabelsi et al., 2012a)	19 physically active men (n = 10, FAST; n = 9, FED)	FAST 26.6 ± 3.0 yrs.<br><br>FED 27.6 ± 1.8 yrs.	RCT	Ramadan Intermittent Fasting.	Abstain from food and drink between dawn and sunset during Ramadan.	Before Ramadan.<br><br>End of Ramadan.	Consuming ad libitum every day.	Before Ramadan.<br><br>End of Ramadan.	Anthropometry	Significant ↓in BW (1.5kg) in FAST group and ↓in BW (2.1kg) in FED group at the end of Ramadan.	d.s	d.s	Significant ↓in FM (1.2%) at the end of Ramadan in FAST group.	No significant changes in both groups.
(Aloui et al., 2013)	12 healthy amateur soccer players.	20.1 ± 1.6 yrs	Observational design	Ramadan Intermittent Fasting.	Abstain from food and drink between dawn	Before and after Ramadan	Consuming ad libitum every day.	1st wk before Ramadan.	Bioimpedance	Significant ↓in BW (0.4kg) at the 4th w of	d.s	d.s	d.s	d.s

					and sunset during Ramadan.			2nd wk of Ramadan. 4th wk of Ramadan. 2 wks after Ramadan.		Ramadan in the afternoon.				
(Norouzy et al., 2013)	240 adults (n = 82, ≤ 35 yrs; n = 158, 36-70 yrs)	18-70 yrs	Observational design	Ramadan Intermittent Fasting.	Abstain from food and drink between dawn and sunset during Ramadan.	Before and after Ramadan.	Consuming ad libitum every day.	Before Ramadan. End of Ramadan.	Bioimpedance	Significant ↓in BW (1.7kg) after Ramadan.	Significant ↓in BMI (0.5kg) after Ramadan.	Significant ↓in FM (0.9kg) after Ramadan.	Significant ↓in FM (0.7%) after Ramadan.	Significant ↓in FFM (1.2kg) after Ramadan.
(Rohin et al., 2013)	46 healthy Muslim adults (n = 14 males; n = 32 females)	33 ± 4.57 yrs	Observational design	Ramadan Intermittent Fasting.	Abstain from food and drink between dawn and sunset during Ramadan.	Before and after Ramadan.	Consuming ad libitum every day.	Before Ramadan. 1st wk of Ramadan. 3rd week of Ramadan. End of Ramadan.	Bioimpedance	Significant ↓in BW after Ramadan.	d.s	d.s	No significant changes in %FM after Ramadan.	d.s
(Trabelsi et al., 2013)	16 male bodybuilders (n = 8, FAST; n = 8, FED)	FAST 25 ± 3 yrs FED 25 ± 2 yrs	RCT	Ramadan Intermittent Fasting.	Abstain from food and drink between dawn and sunset during Ramadan.	Before Ramadan. End of Ramadan.	Consuming ad libitum every day.	Before Ramadan. End of Ramadan.	Anthropometry	BW remained unchanged in FAST and FED group.	BMI remained unchanged in FAST and FED group.	d.s	FM remained unchanged in FAST and FED group.	FFM remained unchanged in FAST and FED group.

(Teng et al., 2013)	56 men (FCR, n = 28; CG, n = 28)	50-70 yrs	RCT	TRF (FCR)	Reduction of 300–500 kcal/d from participants baseline energy intake combined with 2 d/wk of Muslim Sunnah fasting.	CG	Maintain regular food habits.	12 wks	Bioimpedance	Significant ↓ in BW in TRF group.	Significant ↓ in BMI in TRF group.	Significant ↓ in FM in TRF group.	Significant ↓ in relative FM in TRF group.	Significant ↓ in FFM in TRF group.
(Bhutani et al., 2013)	64 Obese subjects (16 for each group – ADF; ADF + Ex; Ex; GC)	40-51 yrs	RCT	ADF<br><br>ADF + Ex	25% of their baseline energy needs on the fast day (24 h), ad libitum on feed day (24h). 12 p.m. to 2 p.m. meals on fast day, (450kcal in each 3 d/wk).	CG	Maintain regular food hgabeabits.	12 wks	Bioimpedance	Significant ↓ in BW in ADF group (3kg) and ADF + Ex group (6kg).	Significant ↓ in BMI in ADF group (1kg) and ADF + Ex group (2kg).	Significant ↓ in FM in ADF group (2kg) and ADF + Ex group (5kg).	d.s	Significant ↓ in FFM in ADF group (1kg).
(Klempel et al., 2013)	32 obese subjects (ADF-LF, n = 17; ADF-HF, n = 15)	ADF-LF 43.2 ± 2.3 yrs<br><br>ADF-HF 42.4 ± 3.0 yrs	RCT	ADF (LF and HF)	25% of their energy needs on the fast day (24h).<br>12 p.m. to 2 p.m. meals on fast day.<br><br>ADF-LF (25% fat).<br><br>ADF-HF (45% fat).	CG	125% of their energy needs on the feed day (24h).	8 wks	DXA	d.s	d.s	Significant ↓ in FM in ADF-LF group (4.2kg) and ADF-HF group (5.4kg).	d.s	FFM remained unchanged.
(Varady et al., 2013)	30 subjects (ADF, n = 15; CG, n = 15).	35-65 yrs	RCT	ADF	25% (400 - 600 kcal) of their baseline energy needs on the fast day (24 h), ad libitum on feed day (24h), 12 p.m. to 2 p.m. meals on fast day.	ND	Ad libitum	12 wks	Bioimpedance	Significant ↓ in BW in ADF group (5.2kg).	d.s	Significant ↓ in FM in ADF group (3.6kg).	d.s	FFM did not change.
(Harvie et al., 2013)	115 overweight women	20-69 yrs	RCT	IECR<br>IECR + PF	≈600 kcal for 2d/wk and	DER	≈1434 kcal 7d/wk	12 wks	Bioimpedance	No significant	d.s	Significant ↓ in FM in	d.s	No significant

	(IECR, n = 37; IECR+PF, n = 38; DER, n = 40)				euenergetic Mediterranean-type diet 5 d/wk.					changes between groups.		IECR (3.7kg) compared with DER group.		changes between groups.
(Keogh et al., 2014)	36 overweight / obesity (CER, n = 17; IER, n = 19)	48-72 yrs	RCT	IER (WOWO)	1wk 'normal' diet followed by 1wk energy restriction (1314kcal).	CER	1314kcal CER	8 wks	Anthropometry	No significant changes between groups.	No significant changes between groups.	d.s	d.s	d.s
(Schoenfeld et al., 2014)	20 healthy young females<br><br>(FAST, n = 10; FED, n = 10)	FAST 23.8 ± 3.0 yrs<br><br>FED 21 ± 1.7	RCT	TRF<br><br>(1236 ± 177Kcal/d)	Performed exercise after an overnight fast.	CG	Performed exercise after consumed a meal (Shake with 250Kcal).<br><br>(1277 ± 137 Kcal/d)	4 wks	Air displacement plethysmography	Significant ↓ in BW in both groups.	Significant ↓ in BMI in both groups.	Significant ↓ in FM in both groups.	No significant changes in both groups.	No significant changes in both groups.
(Abedelmalek et al., 2015)	11 male soccer players	22.1 ± 0.2 yrs	Observational design	Ramadan Intermittent Fasting.	Abstain from food and drink between dawn and sunset during Ramadan.	Before and after Ramadan.	Consuming ad libitum every day.	Before Ramadan.<br><br>1st wk of Ramadan.<br><br>4th wk of Ramadan.	Bioimpedance	Significant ↓in BW (4.2kg) at the end of Ramadan.	Significant ↓in BMI (1.3kg) at the end of Ramadan.	Significant ↓in FM (2.45kg) at the end of Ramadan.	d.s	d.s
(Ismail et al., 2015)	140 overweight or obese Muslim Malay women<br><br>(EG, n = 56; CG, n = 84)	EG 36.65 ± 10.16 yrs<br><br>CG 39.84 ± 10.28 yrs	Non-Randomized control trial	Ramadan Intermittent Fasting.	Abstain from food and drink between dawn and sunset during Ramadan.<br><br>Faith-based dietary intervention promoting voluntary fasting.	Before Ramadan.<br><br>3 months post-Ramadan.	Standard dietary intervention according to national dietary guidelines.	Before Ramadan.<br><br>3 months post-Ramadan.	Anthropometry	d.s	Significant ↓in BMI in EG 3 months after Ramadan compared with CG.	d.s	d.s	d.s

(Roy and Bandyopadhyay, 2015)	77 untrained Muslim men<br><br>(EG, n = 37; CG, n = 40)	EG 22.6 ± 1.8 yrs<br><br>CG 23.0 ± 1.5 yrs	Quasi-experimental design	Ramadan Intermittent Fasting.	Abstain from food and drink between dawn and sunset during Ramadan.	Before and after Ramadan.	CG maintained their normal daily activities including food and water intake.	1 wk before Ramadan.<br><br>During Ramadan.<br><br>4th wk of Ramadan.<br><br>2 wks after Ramadan.	Anthropometry	BW remained unchanged in EG and CG.	d.s	d.s	d.s	d.s
(Varady et al., 2016)	121 obese subjects	50-59 yrs	Non-Randomized control trial	ADF	25% of their baseline energy needs on the fast day (24 h), ad libitum on feed day (24 h).	ND	Ad libitum	8 wks	DXA	Significant ↓ in BW in ADF group.	No significant changes.	d.s	d.s	d.s
(Catenacci et al., 2016)	26 obese participants<br><br>(EG, n = 14; CG, n = 12)	18–55 yrs	RCT	ADF	Fast on alternate days.<br><br>On fed days can eat *at libitum*.	CR	400 kcal/d deficit from estimated energy requirements.	8 wks	DXA	Significant ↓in BW in EG and CG (8.2kg and 7.1kg respectively)	Significant ↓in BMI in EG and CG (3.2kg and 2.4kg respectively)	Significant ↓in FM (3.7kg) in EG and CG.	Significant ↓in FM in EG and CG (1.1% and 1.0% respectively)	Significant ↓in FFM in EG and CG (3.2kg and 2.6kg respectively)
(Moro et al., 2016)	34 resistance-trained males<br><br>(TRF, n = 17; ND, n = 17)	TRF 29.94 ± 4.07<br><br>ND 28.47 ± 3.48	RCT	TRF	16 h per 24-h period made up the fasting period.<br><br>1 p.m., 4 p.m., 8 p.m. feeding.	ND	100 % of their energy needs.	8 wks	DXA	No significant changes in bothgroups.	d.s	Significant ↓in FM (1.62kg) in TRF group.	d.s	FFM was maintained in both groups.
(Syam et al., 2016)	43 medical staff members	34.19 ± 11.25 yrs	Observational design	Ramadan Intermittent Fasting.	Abstain from food and drink between dawn and sunset during Ramadan.	Before and after Ramadan.	Consuming ad libitum every day.	1st d of Ramadan.<br><br>28th d of Ramadan.	Bioimpedance	Significant ↓in BW (0.87kg) at 28th d.	Significant ↓in BMI (0.36kg) at 28th d.	Significant ↓in FM (0.48kg) at 28th d.	d.s	d.s

								4-5 wks after Ramadan.						
(Alsubheen et al., 2017)	16 healthy adult men<br><br>(FAST, n = 8; CG, n = 8)	FAST 32.2 ± 7.8 yrs<br><br>CG 35.0 ± 9.4 yrs	Non-Randomized control trial	Ramadan Intermittent Fasting.	Abstain from food and drink between dawn and sunset during Ramadan.	Before Ramadan.<br><br>4th wk of Ramadan.<br><br>Post-Ramadan.	Maintain regular food habits every day during Ramadan.	Before Ramadan.<br><br>4th wk of Ramadan.<br><br>Post-Ramadan.	Bioimpedance	Significant ↓in BW (2.1kg) post-Ramadan in FAST group.	Significant ↓in BMI (0.6kg) post-Ramadan in FAST group.	Significant ↓in FM (1.4kg) post-Ramadan in FAST group.	Significant ↓in FM (0.8%) post-Ramadan in FAST group.	FFM was maintained in both groups.
(Harder-Lauridsen et al., 2017)	10 healthy, lean men	18-35 yrs	Quasi-experimental design	Ramadan Intermittent Fasting.	Abstain from food and drink between dawn and sunset during Ramadan.	4 wks control period.<br><br>(participants were their own controls)	Maintain regular food and exercise habits.	Before Ramadan.<br><br>After 28th d of Ramadan.	DXA	Slight but significant ↓in BW (1.1kg) after 28 d of Ramadan.	Slight but significant ↓in BMI (0.3kg) after 28 d of Ramadan.	d.s	No significant changes.	No significant changes.
(Tinsley et al., 2017)	18 young recreationally active males<br><br>(TRF, n = 10; ND, n = 8)	TRF 22.9 ± 4.1 yrs<br><br>ND 22.0 ± 2.4 yrs	RCT	TRF	20-h per 24-h period made up the fasting period.<br><br>4-h/d for 4 d/wk consuming ad libitum (4 p.m. and midnight).	ND	Maintain regular normal diet.	8 wks	DXA	No significant changes in BW in both groups.	No significant changes in BMI in both groups.	No significant changes in absolute FM in both groups.	No significant changes in relative FM in both groups.	d.s
(Widhalm et al., 2017)	9 overweight young adults	30-48 yrs	Non-Randomized control trial	ADF	24h of fast on alternate days.	ND	Maximum of 2 successive eating days of ND.	12 wks	DXA	Significant ↓in BW (7.2kg) after 12 wks.	Significant ↓in BMI after 12 wks.	d.s	Significant ↓ FM (11.7%) after 12 wks.	No significant ↓in FFM after 12 wks.
(Trepanowski et al., 2017)	69 healthy obese adults (59 women and 10 men)	18-64 yrs	RCT	ADF	Alternating every 24 h between consuming 25% (between 12 p.m. and 2 p.m.)	DCR	Consuming 75% of needs every day	24 wks	DXA	No significant changes between groups.	No significant changes between groups.	No significant changes between groups.	d.s	No significant changes between groups.

					or 125% of energy needs.									
(Bowen et al., 2018)	135 overweight / obesity (DER, n = 68; ADF + DER, n = 67)	40 ± 8 yrs	RCT	ADF + DER	M, W, F 1195kcal;<br><br>Tu, Th, Su 574kcal;<br>Sa, ad libitum.	DER	Daily energy restriction (1195kcal)	16 wks	DXA	Significant ↓ in BW in ADF + DER group (10kg) and DER group (12.5kg).	Significant ↓ in BMI in ADF + DER group (3.8kg) and DER group (4.4kg).	Significant ↓ in FM in ADF + DER group (8.4kg) and DER group (10.3kg).	d.s	Significant ↓ in FFM in ADF + DER group (1.4kg) and DER group (1.9kg).
(Trepanowski et al., 2018)	79 overweight / obesity<br><br>(ADF, n = 25; CR, n = 29; CG, n = 25)	42-48 yrs	RCT	ADF	Alternating every 24 h between consuming 25% (500kcal) or 125% (2500kcal) of energy needs.	ND<br><br>CR	Consuming 100% of needs every day.<br><br>Consuming 75% (1500kcal) of needs every day.	24 wks	DXA	No significant changes between groups.	d.s	Significant ↓ in FM in ADF group (3kg) compared to CG.	Significant ↓ in FM in ADF group (10%) compared to CG.	No significant changes between groups.
(Akkoca et al., 2018)	80 healthy volunteers	19-50 yrs	Observational design	Ramadan Intermittent Fasting.	Abstain from food and drink between dawn and sunset during Ramadan.	Before and after Ramadan.	Consuming ad libitum every day.	Before Ramadan.<br><br>27th d at the end of Ramadan.	Bioimpedance	Significant ↓ in BW (1.2kg) after 27 d of Ramadan.	Significant ↓ in BMI (0.4kg) after 27 d of Ramadan.	d.s	d.s	d.s
(Gabel et al., 2018)	46 obese subjects<br><br>(TRF, n = 23; CG, n = 23)	TRF 50±2 yrs<br><br>CG 48 ±2 yrs	Non-Randomized control trial	TRF	16 h per 24-h period made up the fasting period.<br><br>Ad libitum feeding between 10 am to 6pm.	ND	Not to change their eating or physical activity habits.	12 wks	DXA	Significant ↓ in BW (3kg) in TRF group.	Significant ↓ in BMI (1kg) in TRF group.	No significant changes in FM in both groups.	d.s	No significant changes in FFM in both groups.
(Lessan et al., 2018)	29 healthy non-obese volunteers (13 men and 16 women)	19-52 yrs	Observational design	Ramadan Intermittent Fasting.	Abstain from food and drink between dawn and sunset during Ramadan.	Before and after Ramadan.	Free-living conditions.	Before Ramadan.<br><br>During Ramadan.	Bioimpedance	No significant changes in BW after Ramadan.	No significant changes in BMI after Ramadan.	No significant changes in absolute FM after Ramadan.	d.s	No significant changes in FFM after Ramadan.

								After Ramadan .						
(Muhammad et al., 2018)	45 overweight/obese subjects<br><br>(13 male and 32 female)	21-56 yrs	Non-Randomized control trial	Ramadan Intermittent Fasting.	Abstain from food and drink between dawn and sunset during Ramadan.	Before and after Ramadan.	Consuming ad libitum every day.	Before Ramadan .<br><br>4th wk of Ramadan .<br><br>6th wk of Ramadan .	Bioimpedance	Significant ↓ in BW (1.4kg) at the end of Ramadan.	Significant ↓ in BMI (0.6kg) at the end of Ramadan.	d.s	Significant ↓ in FM (1.1%) at the end of Ramadan.	No significant changes in FFM at the end of Ramadan.
(Naharudin and Yusof, 2018)	20 healthy active male students	FAS 21 ± 1 yrs<br><br>CG 20 ± 1 yrs	RCT	FAST group	40% energy restriction - omission of lunch.	CG	2492 ± 20 kcal/day	10 days	Anthropometry	Significant ↓ in BW in FAST group.	d.s	d.s	d.s	d.s
(Vargas et al., 2018)	12 women practicing physical activity<br><br>(IF, n = 8; LC, n = 4)	21-47 yrs	Non-Randomized control trial	TRF	4:3 (4d/wk feeding ad libitum, and 3d/wk fasting during 16h).	LC	80g CHO, and the other macronutrients were freely consumed.	8 wks	Anthropometry	Significant ↓ in BW (1.46kg) in TRF group.	d.s	d.s	Significant ↓ in FM (1.63%) in TRF group.	d.s
(Antoni et al., 2018)	27 overweight / obesity (IER, n = 15; CER, n = 12)	IER 42 ± 4 yrs<br><br>CER 48 ± 3 yrs	RCT	IER (5:2)	25% (630 kcal) of their daily recommended energy intake on 2 consecutive d/wk, 5 d/wk of self-selected healthy diet.	CER	Daily hypoenergetic diet (≈ 600kcal)	12 wks	Bioimpedance	Significant ↓ in BW in IER (4.7kg) and CER group (4.4kg).	d.s	Significant ↓ in FM in IER (3.8kg) and CER group (3.8kg).	d.s	Significant ↓ in FFM in IER (1kg) and CER group (0.7kg).
(Nachvak et al., 2019)	152 healthy men	21-63 yrs	Observational design	Ramadan Intermittent Fasting.	Abstain from food and drink between dawn and sunset during Ramadan.	Before and after Ramadan.	Consuming ad libitum every day.	Before Ramadan .<br><br>End of Ramadan .<br><br>1 month after	Bioimpedance	Significant ↓ in BW (2.1kg) at the end of Ramadan.	Significant ↓ in BMI (0.7kg) at the end of Ramadan.	Significant ↓ in FM (0.7kg) at the end of Ramadan.	Significant ↓ in FM (0.37%) at the end of Ramadan.	Significant ↓ in FFM (1.4kg) at the end of Ramadan.

								Ramadan.						
(Headland et al., 2019)	332 overweight / obesity (CER, n = 104; WOWO, n = 110; 5:2, n = 118)	18-72 yrs	RCT	IER (WOWO or 5:2)	WOWO - 1wk of CER and 1wk of habitual diet.<br><br>5:2 - 25% (≈600 kcal) of their daily recommended energy intake on 2 consecutive or non-consecutive d/wk, 5 d/wk of habitual intake.	CER	≈1004 kcal/day for women and ≈1205 kcal/day for men.	8 wks	DXA	Significant ↓ in BW in each group.<br><br>No significant differences between groups.	Significant ↓ in BMI in each group.<br><br>No significant differences between groups.	Significant ↓ in FM in each group.<br><br>No significant differences between groups.	Significant ↓ in relative FM in each group.<br><br>No significant differences between groups.	Significant ↓ in FFM in each group.<br><br>No significant differences between groups.
(Al-Barha and Aljaloud, 2019)	44 healthy university students	18-39 yrs	Non-Randomized control trial	Ramadan Intermittent Fasting.	Abstain from food and drink between dawn and sunset during Ramadan.	Before and after Ramadan.	Consuming ad libitum every day.	Before Ramadan.<br><br>End of the 2nd wk.<br><br>End of the 3th wk.<br><br>6 wks after Ramadan.	Bioimpedance	No significant changes in BW after Ramadan.	No significant changes in BMI after Ramadan.	No significant changes in absolute FM after Ramadan.	No significant changes in relative FM after Ramadan.	No significant changes in FFM after Ramadan.
(Kocaaga et al., 2019)	33 healthy young males	21.85 ± 1.87 yrs	Observational design	Ramadan Intermittent Fasting.	Abstain from food and drink between dawn and sunset during Ramadan.	Before and after Ramadan.	Consuming ad libitum every day.	Before Ramadan.<br><br>1st wk of Ramadan.<br><br>4th wk of Ramadan.	Bioimpedance	Significant ↓ in BW (0.8kg) at the end of Ramadan.	d.s	d.s	Significant ↓ in FM (1.32%) at the end of Ramadan.	No significant changes in FFM at the end of Ramadan.

(Tinsley et al., 2019)	24 healthy women<br><br>(TRF, n = 8; TRF_{HMB}, n = 7; CD, n = 9)	18-30 yrs	RCT	TRF<br><br>TRF_{HMB}	16 h per 24-h period made up the fasting period.<br><br>Ad libitum feeding between 12 am to 8 pm	CD	Consuming regularly from breakfast until the end of the day.	8 wks	DXA	No significant changes in BW after 8wks in TRF group.	d.s	d.s	Significant ↓ in FM in TRF (4%) and TRF_{HMB} (7%).	Significant ↑ in FFM in TRF group.
(Cho et al., 2019)	22 overweight / obesity<br><br>(ADF, n = 8; ADF + Ex, n = 9; CG, n = 5)	28-52 yrs	RCT	ADF<br><br>ADF + Ex	25% (≈ 500 kcal) of their daily recommended energy intake on the fast day (24 h), ad libitum on feed day (24h), 12 p.m. to 2 p.m. meals on fast day.	CG	Ad libitum	8 wks	Bioimpedance	Significant ↓ in BW in ADF group (3.7kg) and ADF + Ex group (3.9kg).	Significant ↓ in BMI in ADF group (1.4kg) and ADF + Ex group (1.5kg).	Significant ↓ in FM in ADF group (2.9kg) and ADF + Ex group (3.1kg).	Significant ↓ in FM in ADF group (2.8%) and ADF + Ex group (2.8%).	d.s
(Kalam et al., 2019)	31 adults with obesity	48 ± 2 yrs	Non-Randomized control trial	ADF	600 kcal on the fast day (24h), alternated with ad libitum on feed day (24h).	Feed day	Ad libitum (1000kcal)	12 wks	DXA	Significant ↓ in BW in ADF group (5.5kg).	d.s	Significant ↓ in FM in ADF group (4kg).	d.s	Significant ↓ in FFM in ADF group (1kg).
(Beaulieu et al., 2020)	30 women with overweight / obesity (CER, n = 18; IER, n = 12)	18-55 yrs	RCT	IER	Alternating ad libitum and 25% of their daily energy requirements.	CER	75% of their daily energy requirements each day.	12 wks	Air displacement plethysmography	Significant ↓ in BW in each group.<br><br>No significant differences between groups.	Significant ↓ in BMI in each group.<br><br>No significant differences between groups.	Significant ↓ in FM in each group.<br><br>No significant differences between groups.	Significant ↓ in relative FM in each group.<br><br>No significant differences between groups.	Significant ↓ in FFM in each group.<br><br>No significant differences between groups.
(Headland et al., 2020)	146 overweight / obesity (CER, n = 53; WOWO, n = 44; 5:2, n = 31)	18-72 yrs	RCT	IER (WOWO or 5:2)	WOWO - 1wk of CER and 1wk of habitual diet.<br><br>5:2 - 25% (≈600 kcal) of their daily recommended energy intake on	CER	1000 kcal/d for women and 1200 kcal/d for men.	8 wks	DXA	Significant ↓ in BW in each group.<br><br>No significant differences between	Significant ↓ in BMI in each group.<br><br>No significant differences IER and CER.	Significant ↓ in FM in each group.<br><br>No significant differences between	d.s	Significant ↓ in FFM in each group.<br><br>No significant differences between IER and CER

					2 consecutive or non-consecutive d/wk, 5 d/wk of habitual intake.					IER and CER		IER and CER		
(Chow et al., 2020)	20 participants with overweight or obesity (TRE, n = 11; non-TRE, n = 9)	TRE 46.5 ± 12.4 yrs<br><br>non-TRE 44.2 ± 12.3 yrs	RCT	TRE	8-hour eating window for ad libitum intake (10:40 am to 6:40 pm or 12 am to 20 pm).	non-TRE	Eat ad libitum per their usual habits.	12 wks	DXA	Significant ↓ in BW (3.6kg) in TRE group.	d.s	Significant ↓ in FM (1.7kg) in TRE group.	Significant ↓ in FM (4%) in TRE group.	Significant ↓ in FFM (1.4kg) in TRE group.

Abbreviations: CD, control diet; CER, continuous energy restriction; CG, control group; CHO, carbohydrate; CR, caloric restriction; DCR, daily calorie restriction; DER, daily energy restriction; d.s, did not say; DXA, dual x-ray absorptiometry; Ex, exercise; F, Friday; FCR, fasting calorie restriction; FFM, fat-free mass; FM, fat mass; HL, hight fat; HMB, β-hydroxy β-methylbutyrate; IECR, intermittent energy and carbohydrate restriction; IECR + PF, IECR which allowed ad libitum protein and fat; Kcal, Calories; LC, low carb diet; LF, low fat; M, Monday; ND, normal diet; Sa, Saturday; Su, Sunday; Th, Thursday; TRE, time-restricted eating; TRF, time-restricted feeding; TRFHMB, TRF plus 3 g/d HMB; Tu, Tuesday; VLCD, very low calorie diet; W, Wednesday; WOWO, week-on-week-off.

References

Abedelmalek, S., Denguezli, M., Chtourou, H., Souissi, N., and Tabka, Z. (2015). Does Ramadan fasting affect acylated ghrelin and growth hormone concentrations during short-term maximal exercise in the afternoon? *Biol Rhythm Res* 46(5), 691-701. doi: 10.1080/09291016.2015.1048949.

Akkoca, M., Metin, Z.E., Topaloglu, O., Tokgoz, S., Cihan, G., and San, I. (2018). An evaluation of the effects of Ramadan fasting on anthropometric, metabolic and endocrine parameters. *Prog Nutr* 20(3), 503-509. doi: 10.23751/pn.v20i3.7588.

Al-Barha, N.S., and Aljaloud, K.S. (2019). The Effect of Ramadan Fasting on Body Composition and Metabolic Syndrome in Apparently Healthy Men. *Am J Mens Health* 13(1), 1557988318816925. doi: 10.1177/1557988318816925.

Al-Hourani, H.M., and Atoum, M.F. (2007). Body composition, nutrient intake and physical activity patterns in young women during Ramadan. *Singapore Med J* 48(10), 906-910.

Al-Numair, K. (2006a). Body weight and some biochemical changes associated with Ramadan fasting in healthy Saudi men. *J Med Sci* 57(1), 67.

Al-Numair, K. (2006b). Body Weight and Some Biochemical Changes Associated with Ramadan Fasting in Healthy Saudi Men. *J Med Sci* 6(1), 112-116. doi: 10.3923/jms.2006.112.116.

Aloui, A., Chaouachi, A., Chtourou, H., Wong, D.P., Haddad, M., Chamari, K., et al. (2013). Effects of Ramadan on the diurnal variations of repeated-sprint performance. *Int J Sports Physiol Perform* 8(3), 254-263.

Alsubheen, S.A., Ismail, M., Baker, A., Blair, J., Adebayo, A., Kelly, L., et al. (2017). The effects of diurnal Ramadan fasting on energy expenditure and substrate oxidation in healthy men. *Br J Nutr* 118(12), 1023-1030. doi: 10.1017/S0007114517003221.

Anton, S.D., Moehl, K., Donahoo, W.T., Marosi, K., Lee, S.A., Mainous, A.G., 3rd, et al. (2018). Flipping the Metabolic Switch: Understanding and Applying the Health Benefits of Fasting. *Obesity (Silver Spring)* 26(2), 254-268. doi: 10.1002/oby.22065.

Antoni, R., Johnston, K.L., Collins, A.L., and Robertson, M.D. (2018). Intermittent v. continuous energy restriction: differential effects on postprandial glucose and lipid metabolism following matched weight loss in overweight/obese participants. *Br J Nutr* 119(5), 507-516. doi: 10.1017/S0007114517003890.

Beaulieu, K., Casanova, N., Oustric, P., Turicchi, J., Gibbons, C., Hopkins, M., et al. (2020). Matched weight loss through intermittent or continuous energy restriction does not lead to compensatory increases in appetite and eating behavior in a randomized controlled trial in women with overweight and obesity. *Nutr J* 150(3), 623-633.

Beltaifa, L., Bouguerra, R., Ben Slama, C., Jabrane, H., El-Khadhi, A., Ben Rayana, M.C., et al. (2002). [Food intake, and anthropometrical and biological parameters in adult Tunisians during fasting at Ramadan]. *East Mediterr Health J* 8(4-5), 603-611.

Bhutani, S., Klempel, M.C., Kroeger, C.M., Trepanowski, J.F., and Varady, K.A. (2013). Alternate day fasting and endurance exercise combine to reduce body weight and favorably alter plasma lipids in obese humans. *Obesity (Silver Spring)* 21(7), 1370-1379. doi: 10.1002/oby.20353.

Bilto, Y.Y. (1998). Effects of Ramadan fasting on body weight and the biochemical and haematological parameters of the blood. *Arab Gulf J Sci Res* 16(1), 1-13.

Borenstein, M., Hedges, L., and Higgins, J. (2009a). Rothstein HR. Introduction to meta-analysis. *West Sussex, England: Wiley & Sons Ltd*.

Borenstein, M., Hedges, L., Higgins, J., and Rothstein, H. (2005). Comprehensive meta-analysis version 2. *Englewood, NJ: Biostat* 104.

Borenstein, M., Hedges, L.V., Higgins, J.P., and Rothstein, H.R. (2009b). "Meta-analysis methods based on direction and p-values. Introduction to Meta-Analysis". *Chichester, UK: John Wiley & Sons*.

Bowen, J., Brindal, E., James-Martin, G., and Noakes, M. (2018). Randomized trial of a high protein, partial meal replacement program with or without alternate day fasting: similar effects on weight loss, retention status, nutritional, metabolic, and behavioral outcomes. *Nutrients* 10(9), 1145.

Catenacci, V.A., Pan, Z., Ostendorf, D., Brannon, S., Gozansky, W.S., Mattson, M.P., et al. (2016). A randomized pilot study comparing zero-calorie alternate-day fasting to daily caloric restriction in adults with obesity. *Obesity (Silver Spring)* 24(9), 1874-1883. doi: 10.1002/oby.21581.

Cava, E., Yeat, N.C., and Mittendorfer, B. (2017). Preserving Healthy Muscle during Weight Loss. *Adv Nutr* 8(3), 511-519. doi: 10.3945/an.116.014506.

Chennaoui, M., Desgorces, F., Drogou, C., Boudjemaa, B., Tomaszewski, A., Depiesse, F., et al. (2009). Effects of Ramadan fasting on physical performance and metabolic, hormonal, and inflammatory parameters in middle-distance runners. *Appl Physiol Nutr Metab* 34(4), 587-594. doi: 10.1139/H09-014.

Cho, A.R., Moon, J.Y., Kim, S., An, K.Y., Oh, M., Jeon, J.Y., et al. (2019). Effects of alternate day fasting and exercise on cholesterol metabolism in overweight or obese adults: A pilot randomized controlled trial. *Metabolism* 93, 52-60. doi: 10.1016/j.metabol.2019.01.002.

Chow, L.S., Manoogian, E.N.C., Alvear, A., Fleischer, J.G., Thor, H., Dietsche, K., et al. (2020). Time-Restricted Eating Effects on Body Composition and Metabolic Measures in Humans who are Overweight: A Feasibility Study. *Obesity (Silver Spring)* 28(5), 860-869. doi: 10.1002/oby.22756.

Cochran, W.G. (1954). The Combination of Estimates from Different Experiments. *Biometrics* 10(1), 101-129. doi: Doi 10.2307/3001666.

Cohen, J. (1992). A power primer. *Psychol Bull* 112(1), 155-159.

Davis, C.S., Clarke, R.E., Coulter, S.N., Rounsefell, K.N., Walker, R.E., Rauch, C.E., et al. (2016). Intermittent energy restriction and weight loss: a systematic review. *Eur J Clin Nutr* 70(3), 292-299. doi: 10.1038/ejcn.2015.195.

De Bock, K., Derave, W., Eijnde, B.O., Hesselink, M.K., Koninckx, E., Rose, A.J., et al. (2008). Effect of training in the fasted state on metabolic responses during exercise with carbohydrate intake. *J Appl Physiol (1985)* 104(4), 1045-1055. doi: 10.1152/japplphysiol.01195.2007.

Duval, S., and Tweedie, R. (2000). Trim and fill: a simple funnel-plot–based method of testing and adjusting for publication bias in meta-analysis. *Biometrics* 56(2), 455-463.

el Ati, J., Beji, C., and Danguir, J. (1995). Increased fat oxidation during Ramadan fasting in healthy women: an adaptative mechanism for body-weight maintenance. *Am J Clin Nutr* 62(2), 302-307. doi: 10.1093/ajcn/62.2.302.

Ferguson, L.M., Rossi, K.A., Ward, E., Jadwin, E., Miller, T.A., and Miller, W.C. (2009). Effects of caloric restriction and overnight fasting on cycling endurance performance. *J Strength Cond Res* 23(2), 560-570. doi: 10.1519/JSC.0b013e31818f058b.

Gabel, K., Hoddy, K.K., Haggerty, N., Song, J., Kroeger, C.M., Trepanowski, J.F., et al. (2018). Effects of 8-hour time restricted feeding on body weight and metabolic disease risk factors in obese adults: A pilot study. *Nutr Healthy Aging* 4(4), 345-353. doi: 10.3233/NHA-170036.

Hajek, P., Myers, K., Dhanji, A.R., West, O., and McRobbie, H. (2012). Weight change during and after Ramadan fasting. *J Public Health* 34(3), 377-381. doi: 10.1093/pubmed/fdr087.

Haouari, M., Haouari-Oukerro, F., Sfaxi, A., Ben Rayana, M.C., Kaabachi, N., and Mbazaa, A. (2008). How Ramadan fasting affects caloric consumption, body weight, and circadian evolution of cortisol serum levels in young, healthy male volunteers. *Horm Metab Res* 40(8), 575-577. doi: 10.1055/s-2008-1065321.

Harder-Lauridsen, N.M., Rosenberg, A., Benatti, F.B., Damm, J.A., Thomsen, C., Mortensen, E.L., et al. (2017). Ramadan model of intermittent fasting for 28 d had no major effect on body composition, glucose metabolism, or cognitive functions in healthy lean men. *Nutrition* 37, 92-103. doi: 10.1016/j.nut.2016.12.015.

Harvie, M., and Howell, A. (2017). Potential benefits and harms of intermittent energy restriction and intermittent fasting amongst obese, overweight and normal weight subjects—a narrative review of human and animal evidence. *Behav Sci* 7(1), 4.

Harvie, M., Wright, C., Pegington, M., McMullan, D., Mitchell, E., Martin, B., et al. (2013). The effect of intermittent energy and carbohydrate restriction v. daily energy restriction on weight loss and metabolic disease risk markers in overweight women. *Br J Nutr* 110(8), 1534-1547. doi: 10.1017/S0007114513000792.

Harvie, M.N., Pegington, M., Mattson, M.P., Frystyk, J., Dillon, B., Evans, G., et al. (2011). The effects of intermittent or continuous energy restriction on weight loss and metabolic disease risk markers: a randomized trial in young overweight women. *Int J Obes (Lond)* 35(5), 714-727. doi: 10.1038/ijo.2010.171.

Headland, M.L., Clifton, P.M., and Keogh, J.B. (2019). Effect of intermittent compared to continuous energy restriction on weight loss and weight maintenance after 12 months in healthy overweight or obese adults. *Int J Obes* 43(10), 2028-2036.

Headland, M.L., Clifton, P.M., and Keogh, J.B. (2020). Impact of intermittent vs. continuous energy restriction on weight and cardiometabolic factors: a 12-month follow-up. *Int J Obes (Lond)*, 1-7. doi: 10.1038/s41366-020-0525-7.

Heilbronn, L.K., Smith, S.R., Martin, C.K., Anton, S.D., and Ravussin, E. (2005). Alternate-day fasting in nonobese subjects: effects on body weight, body composition, and energy metabolism. *Am J Clin Nutr* 81(1), 69-73. doi: 10.1093/ajcn/81.1.69.

Higgins, J.P., Thompson, S.G., Deeks, J.J., and Altman, D.G. (2003). Measuring inconsistency in meta-analyses. *BMJ* 327(7414), 557-560. doi: 10.1136/bmj.327.7414.557.

Ismail, S., Shamsuddin, K., Latiff, K.A., Saad, H.A., Majid, L.A., and Othman, F.M. (2015). Voluntary Fasting to Control Post-Ramadan Weight Gain among Overweight and Obese Women. *Sultan Qaboos Univ Med J* 15(1), e98-e104.

Kalam, F., Gabel, K., Cienfuegos, S., Wiseman, E., Ezpeleta, M., Steward, M., et al. (2019). Alternate day fasting combined with a low-carbohydrate diet for weight loss, weight maintenance, and metabolic disease risk reduction. *Obes Sci Pract* 5(6), 531-539. doi: 10.1002/osp4.367.

Karli, U., Guvenc, A., Aslan, A., Hazir, T., and Acikada, C. (2007). Influence of Ramadan Fasting on Anaerobic Performance and Recovery Following Short time High Intensity Exercise. *J Sports Sci Med* 6(4), 490-497.

Keogh, J.B., Pedersen, E., Petersen, K.S., and Clifton, P.M. (2014). Effects of intermittent compared to continuous energy restriction on short-term weight loss and long-term weight loss maintenance. *Clin Obes* 4(3), 150-156. doi: 10.1111/cob.12052.

Klempel, M.C., Kroeger, C.M., and Varady, K.A. (2013). Alternate day fasting (ADF) with a high-fat diet produces similar weight loss and cardio-protection as ADF with a low-fat diet. *Metabolism* 62(1), 137-143.

Kocaaga, T., Tamer, K., Karli, U., and Yarar, H. (2019). Effects of Ramadan Fasting on Physical Activity Level and Body Composition in Young Males. *Int J Appl Exerc Physio* 8(3).

Kreitzman, S.N., Coxon, A.Y., and Szaz, K.F. (1992). Glycogen storage: illusions of easy weight loss, excessive weight regain, and distortions in estimates of body composition. *Am J Clin Nutr* 56(1 Suppl), 292S-293S. doi: 10.1093/ajcn/56.1.292S.

Lessan, N., Saadane, I., Alkaf, B., Hambly, C., Buckley, A.J., Finer, N., et al. (2018). The effects of Ramadan fasting on activity and energy expenditure. *Am J Clin Nutr* 107(1), 54-61. doi: 10.1093/ajcn/nqx016.

Mattson, M.P., Longo, V.D., and Harvie, M. (2017). Impact of intermittent fasting on health and disease processes. *Ageing Res Rev* 39, 46-58. doi: 10.1016/j.arr.2016.10.005.

Mirzaei, B., Rahmani-Nia, F., Moghadam, M.G., Ziyaolhagh, S.J., and Rezaei, A. (2012). The effect of ramadan fasting on biochemical and performance parameters in collegiate wrestlers. *Iran J Basic Med Sci* 15(6), 1215-1220.

Moher, D., Liberati, A., Tetzlaff, J., Altman, D.G., and Group, P. (2009). Reprint—preferred reporting items for systematic reviews and meta-analyses: the PRISMA statement. *Phys Ther* 89(9), 873-880.

Moro, T., Tinsley, G., Bianco, A., Marcolin, G., Pacelli, Q.F., Battaglia, G., et al. (2016). Effects of eight weeks of time-restricted feeding (16/8) on basal metabolism, maximal strength, body composition, inflammation, and cardiovascular risk factors in resistance-trained males. *J Transl Med* 14(1), 290. doi: 10.1186/s12967-016-1044-0.

Muhammad, H.F.L., Latifah, F.N., and Susilowati, R. (2018). The yo-yo effect of Ramadan fasting on overweight/obese individuals in Indonesian: A prospective study. *Med J Nutrition Metab* 11(2), 127-133. doi: 10.3233/mnm-17188.

Nachvak, S.M., Pasdar, Y., Pirsaheb, S., Darbandi, M., Niazi, P., Mostafai, R., et al. (2019). Effects of Ramadan on food intake, glucose homeostasis, lipid profiles and body composition composition. *Eur J Clin Nutr* 73(4), 594-600. doi: 10.1038/s41430-018-0189-8.

Naharudin, M.N.B., and Yusof, A. (2018). The effect of 10 days of intermittent fasting on Wingate anaerobic power and prolonged high-intensity time-to-exhaustion cycling performance. *Eur J Sport Sci* 18(5), 667-676. doi: 10.1080/17461391.2018.1438520.

Norouzy, A., Salehi, M., Philippou, E., Arabi, H., Shiva, F., Mehrnoosh, S., et al. (2013). Effect of fasting in Ramadan on body composition and nutritional intake: a prospective study. *J Hum Nutr Diet* 26 Suppl 1, 97-104. doi: 10.1111/jhn.12042.

Ramadan, J. (2002). Does fasting during Ramadan alter body composition, blood constituents and physical performance? *Med Princ Pract* 11 Suppl 2, 41-46. doi: 10.1159/000066413.

Ramadan, J., Telahoun, G., Al-Zaid, N.S., and Barac-Nieto, M. (1999). Responses to exercise, fluid, and energy balances during Ramadan in sedentary and active males. *Nutrition* 15(10), 735-739. doi: 10.1016/s0899-9007(99)00145-8.

Rohin, M.A., Rozano, N., Abd Hadi, N., Mat Nor, M.N., Abdullah, S., and Dandinasivara Venkateshaiah, M. (2013). Anthropometry and body composition status during Ramadan among higher institution learning centre staffs with different body weight status. *Sci World J,* 308041. doi: 10.1155/2013/308041.

Roy, A.S., and Bandyopadhyay, A. (2015). Effect of Ramadan intermittent fasting on selective fitness profile parameters in young untrained Muslim men. *BMJ Open Sport Exerc Med* 1(1), e000020. doi: 10.1136/bmjsem-2015-000020.

Salehi, M., and Neghab, M. (2007). Effects of fasting and a medium calorie balanced diet during the holy month Ramadan on weight, BMI and some blood parameters of overweight males. *Pak J Biol Sci* 10(6), 968-971.

Schoenfeld, B.J., Aragon, A.A., Wilborn, C.D., Krieger, J.W., and Sonmez, G.T. (2014). Body composition changes associated with fasted versus non-fasted aerobic exercise. *J Int Soc Sports Nutr* 11(1), 54. doi: 10.1186/s12970-014-0054-7.

Sedgwick, P., and Marston, L. (2013). Meta-analyses: standardised mean differences. *Bmj-Brit Med J* 347. doi: Artn F725710.1136/Bmj.F7257.

Stannard, S.R., and Thompson, M.W. (2008). The effect of participation in Ramadan on substrate selection during submaximal cycling exercise. *J Sci Med Sport* 11(5), 510-517. doi: 10.1016/j.jsams.2007.03.003.

Sterne, J.A., and Egger, M. (2001). Funnel plots for detecting bias in meta-analysis: guidelines on choice of axis. *J Clin Epidemiol* 54(10), 1046-1055. doi: 10.1016/s0895-4356(01)00377-8.

Stote, K.S., Baer, D.J., Spears, K., Paul, D.R., Harris, G.K., Rumpler, W.V., et al. (2007). A controlled trial of reduced meal frequency without caloric restriction in healthy, normal-weight, middle-aged adults. *Am J Clin Nutr* 85(4), 981-988.

SÜLÜ, B., ÖZtÜRk, B., GÜVen, A., and KiliÇ, K. (2010). The Effect of Long-Term Controlled Fasting (The Ramadan Model) on Body Mass Index, Blood Biochemistry and Oxidative Stress Factors. *Turk Klin J Med Sci* 30(3), 855-863. doi: 10.5336/medsci.2008-9981.

Syam, A.F., Sobur, C.S., Abdullah, M., and Makmun, D. (2016). Ramadan fasting decreases body fat but not protein mass. *Int J Endocrinol Metab* 14(1).

Templeman, I., Thompson, D., Gonzalez, J., Walhin, J.P., Reeves, S., Rogers, P.J., et al. (2018). Intermittent fasting, energy balance and associated health outcomes in adults: study protocol for a randomised controlled trial. *Trials* 19(1), 86. doi: 10.1186/s13063-018-2451-8.

Teng, N.I., Shahar, S., Rajab, N.F., Manaf, Z.A., Johari, M.H., and Ngah, W.Z. (2013). Improvement of metabolic parameters in healthy older adult men following a fasting calorie restriction intervention. *Aging Male* 16(4), 177-183. doi: 10.3109/13685538.2013.832191.

Thomas, B.H., Ciliska, D., Dobbins, M., and Micucci, S. (2004). A process for systematically reviewing the literature: providing the research evidence for public health nursing interventions. *Worldviews Evid Based Nurs* 1(3), 176-184. doi: 10.1111/j.1524-475X.2004.04006.x.

Tinsley, G.M., Forsse, J.S., Butler, N.K., Paoli, A., Bane, A.A., La Bounty, P.M., et al. (2017). Time-restricted feeding in young men performing resistance training: A randomized controlled trial. *Eur J Sport Sci* 17(2), 200-207. doi: 10.1080/17461391.2016.1223173.

Tinsley, G.M., Moore, M.L., Graybeal, A.J., Paoli, A., Kim, Y., Gonzales, J.U., et al. (2019). Time-restricted feeding plus resistance training in active females: a randomized trial. *Am J Clin Nutr* 110(3), 628-640. doi: 10.1093/ajcn/nqz126.

Trabelsi, K., el Abed, K., Stannard, S.R., Jammoussi, K., Zeghal, K.M., and Hakim, A. (2012a). Effects of fed- versus fasted-state aerobic training during Ramadan on body composition and some metabolic parameters in physically active men. *Int J Sport Nutr Exerc Metab* 22(1), 11-18. doi: 10.1123/ijsnem.22.1.11.

Trabelsi, K., Stannard, S.R., Ghlissi, Z., Maughan, R.J., Kallel, C., Jamoussi, K., et al. (2013). Effect of fed- versus fasted state resistance training during Ramadan on body composition and selected metabolic parameters in bodybuilders. *J Int Soc Sports Nutr* 10(1), 23. doi: 10.1186/1550-2783-10-23.

Trabelsi, K., Stannard, S.R., Maughan, R.J., Jammoussi, K., Zeghal, K., and Hakim, A. (2012b). Effect of resistance training during Ramadan on body composition and markers of renal function, metabolism, inflammation, and immunity in recreational bodybuilders. *Int J Sport Nutr Exerc Metab* 22(4), 267-275. doi: 10.1123/ijsnem.22.4.267.

Trepanowski, J.F., Kroeger, C.M., Barnosky, A., Klempel, M., Bhutani, S., Hoddy, K.K., et al. (2018). Effects of alternate-day fasting or daily calorie restriction on body composition, fat distribution, and circulating adipokines: Secondary analysis of a randomized controlled trial. *Clin Nutr* 37(6 Pt A), 1871-1878. doi: 10.1016/j.clnu.2017.11.018.

Trepanowski, J.F., Kroeger, C.M., Barnosky, A., Klempel, M.C., Bhutani, S., Hoddy, K.K., et al. (2017). Effect of alternate-day fasting on weight loss, weight maintenance, and cardioprotection among metabolically healthy obese adults: a randomized clinical trial. *JAMA Intern Med* 177(7), 930-938.

Van Proeyen, K., Szlufcik, K., Nielens, H., Pelgrim, K., Deldicque, L., Hesselink, M., et al. (2010). Training in the fasted state improves glucose tolerance during fat-rich diet. *J Physiol* 588(Pt 21), 4289-4302. doi: 10.1113/jphysiol.2010.196493.

Varady, K. (2011). Intermittent versus daily calorie restriction: which diet regimen is more effective for weight loss? *Obes Rev* 12(7), e593-e601.

Varady, K.A., Bhutani, S., Klempel, M.C., Kroeger, C.M., Trepanowski, J.F., Haus, J.M., et al. (2013). Alternate day fasting for weight loss in normal weight and overweight subjects: a randomized controlled trial. *Nutr J* 12(1), 146. doi: 10.1186/1475-2891-12-146.

Varady, K.A., Hoddy, K.K., Kroeger, C.M., Trepanowski, J.F., Klempel, M.C., Barnosky, A., et al. (2016). Determinants of weight loss success with alternate day fasting. *Obes Res Clin Pract* 10(4), 476-480. doi: 10.1016/j.orcp.2015.08.020.

Vargas, A.J., Pessoa, L.D., and da Rosa, R.L. (2018). Intermittent fasting and low carb diet in the body composition of women practicing physical activity. *Rev Bras Nutr Esportiva* 12(72), 483-490.

Widhalm, K., Pöppelmeyer, C., and Helk, O. (2017). The Effect of Alternate-Day Fasting (ADF) on Weight Loss, Metabolic Parameters and Psychological Characteristics. *Aktuelle Ernährungsmedizin* 42(03), 188-192. doi: 10.1055/s-0043-109126.

Ziaee, V., Razaei, M., Ahmadinejad, Z., Shaikh, H., Yousefi, R., Yarmohammadi, L., et al. (2006). The changes of metabolic profile and weight during Ramadan fasting. *Singapore Med J* 47(5), 409-414.

Zouhal, H., Saeidi, A., Salhi, A., Li, H., Essop, M.F., Laher, I., et al. (2020). Exercise Training and Fasting: Current Insights. *Open Access J Sports Med* 11, 1-28. doi: 10.2147/OAJSM.S224919.
