# Supplementary material for: Effects of Ramadan and Non-ramadan Intermittent Fasting on Body Composition: A Systematic Review and Meta-Analysis
Source: Front Nutr. 2021 Jan 26;7:625240. doi: 10.3389/fnut.2020.625240 (PMC7870696; doi:10.3389/fnut.2020.625240)
Supplement: Supplementary file 2 [file Table_2.pdf]

Supplementary table. 2 Quality assessment of all included studies.

Reference	Selection Bias	Study Design	Confounders	Data Collection Method	Withdrawals	Gobal
(el Ati et al., 1995)	1	1	3	3	N/A	Weak
(Bilto, 1998)	1	1	3	3	N/A	Weak
(Ramadan et al., 1999)	1	1	3	3	N/A	Weak
(Beltaifa et al., 2002)	1	1	3	3	N/A	Weak
(Ramadan, 2002)	1	1	3	3	N/A	Weak
(Heilbronn et al., 2005)	1	3	3	3	3	Moderate
(Al-Numair, 2006a)	1	1	3	3	N/A	Weak
(Ziaee et al., 2006)	1	1	3	3	N/A	Weak
(Al-Hourani and Atoum, 2007)	1	1	3	3	N/A	Weak
(Karli et al., 2007)	1	1	3	3	N/A	Weak
(Salehi and Neghab, 2007)	1	2	3	3	3	Moderate
(Stote et al., 2007)	1	3	3	3	3	Moderate
(Haouari et al., 2008)	1	1	3	3	N/A	Weak
(De Bock et al., 2008)	1	2	3	3	3	Moderate
(Stannard and Thompson, 2008)	1	1	3	3	N/A	Weak
(Chennaoui et al., 2009)	1	1	3	3	2	Moderate
(Ferguson et al., 2009)	1	2	3	3	3	Moderate
(SÜLÜ et al., 2010)	1	1	3	3	N/A	Weak
(Van Proeyen et al., 2010)	1	3	3	3	3	Moderate
(Harvie et al., 2011)	1	3	3	3	2	Moderate
(Hajek et al., 2012)	1	1	3	3	N/A	Weak
(Mirzaei et al., 2012)	1	1	3	3	N/A	Weak
(Trabelsi et al., 2012b)	1	2	3	3	3	Moderate
(Trabelsi et al., 2012a)	1	3	3	3	3	Moderate
(Aloui et al., 2013)	1	1	3	3	N/A	Weak
(Norouzy et al., 2013)	1	1	3	3	N/A	Weak
(Rohin et al., 2013)	1	1	3	3	N/A	Weak
(Trabelsi et al., 2013)	1	3	3	3	N/A	Moderate
(Teng et al., 2013)	1	3	3	3	3	Moderate
(Bhutani et al., 2013)	1	3	3	3	2	Moderate
(Klempel et al., 2013)	1	3	3	3	2	Moderate
(Varady et al., 2013)	1	3	3	3	3	Moderate
(Harvie et al., 2013)	1	3	3	3	2	Moderate
(Keogh et al., 2014)	1	3	3	3	2	Moderate

(Schoenfeld et al., 2014)	1	3	3	3	3	Moderate
(Abedelmalek et al., 2015)	1	1	3	3	N/A	Weak
(Ismail et al., 2015)	1	2	3	3	2	Moderate
(Roy and Bandyopadhyay, 2015)	1	1	3	3	N/A	Weak
(Varady et al., 2016)	1	2	3	3	3	Moderate
(Catenacci et al., 2016)	1	3	3	3	3	Moderate
(Moro et al., 2016)	1	3	3	3	3	Moderate
(Syam et al., 2016)	1	1	3	3	N/A	Weak
(Alsubheen et al., 2017)	1	2	3	3	3	Moderate
(Harder-Lauridsen et al., 2017)	1	1	3	3	N/A	Weak
(Tinsley et al., 2017)	1	3	3	3	2	Moderate
(Widhalm et al., 2017)	1	2	3	3	2	Moderate
(Trepanowski et al., 2017)	1	3	3	3	2	Moderate
(Bowen et al., 2018)	1	3	3	3	3	Moderate
(Trepanowski et al., 2018)	1	3	3	3	2	Moderate
(Akkoca et al., 2018)	1	1	3	3	N/A	Weak
(Gabel et al., 2018)	1	2	3	3	3	Moderate
(Lessan et al., 2018)	1	1	3	3	N/A	Weak
(Muhammad et al., 2018)	1	2	3	3	2	Moderate
(Naharudin and Yusof, 2018)	1	3	3	3	3	Moderate
(Vargas et al., 2018)	1	2	3	3	1	Weak
(Antoni et al., 2018)	1	3	3	3	2	Moderate
(Nachvak et al., 2019)	1	1	3	3	N/A	Weak
(Headland et al., 2019)	1	3	3	3	2	Moderate
(Al-Barha and Aljaloud, 2019)	1	2	3	3	2	Moderate
(Kocaaga et al., 2019)	1	1	3	3	N/A	Weak
(Tinsley et al., 2019)	1	3	3	3	2	Moderate
(Cho et al., 2019)	1	3	3	3	2	Moderate
(Kalam et al., 2019)	1	2	3	3	2	Moderate
(Beaulieu et al., 2020)	1	3	3	3	3	Moderate
(Headland et al., 2020)	1	3	3	3	2	Moderate
(Chow et al., 2020)	1	3	3	3	3	Moderate

Note. 1, weak; 2, moderate; 3, strong, N/A, not applicable.

References

Abedelmalek, S., Denguezli, M., Chtourou, H., Souissi, N., and Tabka, Z. (2015). Does Ramadan fasting affect acylated ghrelin and growth hormone concentrations during short-term maximal exercise in the afternoon? *Biol Rhythm Res* 46(5), 691-701. doi: 10.1080/09291016.2015.1048949.

Akkoca, M., Metin, Z.E., Topaloglu, O., Tokgoz, S., Cihan, G., and San, I. (2018). An evaluation of the effects of Ramadan fasting on anthropometric, metabolic and endocrine parameters. *Prog Nutr* 20(3), 503-509. doi: 10.23751/pn.v20i3.7588.

Al-Barha, N.S., and Aljaloud, K.S. (2019). The Effect of Ramadan Fasting on Body Composition and Metabolic Syndrome in Apparently Healthy Men. *Am J Mens Health* 13(1), 1557988318816925. doi: 10.1177/1557988318816925.

Al-Hourani, H.M., and Atoum, M.F. (2007). Body composition, nutrient intake and physical activity patterns in young women during Ramadan. *Singapore Med J* 48(10), 906-910.

Al-Numair, K. (2006a). Body weight and some biochemical changes associated with Ramadan fasting in healthy Saudi men. *J Med Sci* 57(1), 67.

Al-Numair, K. (2006b). Body Weight and Some Biochemical Changes Associated with Ramadan Fasting in Healthy Saudi Men. *J Med Sci* 6(1), 112-116. doi: 10.3923/jms.2006.112.116.

Aloui, A., Chaouachi, A., Chtourou, H., Wong, D.P., Haddad, M., Chamari, K., et al. (2013). Effects of Ramadan on the diurnal variations of repeated-sprint performance. *Int J Sports Physiol Perform* 8(3), 254-263.

Alsubheen, S.A., Ismail, M., Baker, A., Blair, J., Adebayo, A., Kelly, L., et al. (2017). The effects of diurnal Ramadan fasting on energy expenditure and substrate oxidation in healthy men. *Br J Nutr* 118(12), 1023-1030. doi: 10.1017/S0007114517003221.

Anton, S.D., Moehl, K., Donahoo, W.T., Marosi, K., Lee, S.A., Mainous, A.G., 3rd, et al. (2018). Flipping the Metabolic Switch: Understanding and Applying the Health Benefits of Fasting. *Obesity (Silver Spring)* 26(2), 254-268. doi: 10.1002/oby.22065.

Antoni, R., Johnston, K.L., Collins, A.L., and Robertson, M.D. (2018). Intermittent v. continuous energy restriction: differential effects on postprandial glucose and

lipid metabolism following matched weight loss in overweight/obese participants. *Br J Nutr* 119(5), 507-516. doi: 10.1017/S0007114517003890.

Beaulieu, K., Casanova, N., Oustric, P., Turicchi, J., Gibbons, C., Hopkins, M., et al. (2020). Matched weight loss through intermittent or continuous energy restriction does not lead to compensatory increases in appetite and eating behavior in a randomized controlled trial in women with overweight and obesity. *Nutr J* 150(3), 623-633.

Beltaifa, L., Bouguerra, R., Ben Slama, C., Jabrane, H., El-Khadhi, A., Ben Rayana, M.C., et al. (2002). [Food intake, and anthropometrical and biological parameters in adult Tunisians during fasting at Ramadan]. *East Mediterr Health J* 8(4-5), 603-611.

Bhutani, S., Klempel, M.C., Kroeger, C.M., Trepanowski, J.F., and Varady, K.A. (2013). Alternate day fasting and endurance exercise combine to reduce body weight and favorably alter plasma lipids in obese humans. *Obesity (Silver Spring)* 21(7), 1370-1379. doi: 10.1002/oby.20353.

Bilto, Y.Y. (1998). Effects of Ramadan fasting on body weight and the biochemical and haematological parameters of the blood. *Arab Gulf J Sci Res* 16(1), 1-13.

Borenstein, M., Hedges, L., and Higgins, J. (2009a). Rothstein HR. Introduction to meta-analysis. *West Sussex, England: Wiley & Sons Ltd*.

Borenstein, M., Hedges, L., Higgins, J., and Rothstein, H. (2005). Comprehensive meta-analysis version 2. *Englewood, NJ: Biostat* 104.

Borenstein, M., Hedges, L.V., Higgins, J.P., and Rothstein, H.R. (2009b). "Meta-analysis methods based on direction and p-values. Introduction to Meta-Analysis". *Chichester, UK: John Wiley & Sons*.

Bowen, J., Brindal, E., James-Martin, G., and Noakes, M. (2018). Randomized trial of a high protein, partial meal replacement program with or without alternate day fasting: similar effects on weight loss, retention status, nutritional, metabolic, and behavioral outcomes. *Nutrients* 10(9), 1145.

Catenacci, V.A., Pan, Z., Ostendorf, D., Brannon, S., Gozansky, W.S., Mattson, M.P., et al. (2016). A randomized pilot study comparing zero-calorie alternate-day fasting to daily caloric restriction in adults with obesity. *Obesity (Silver Spring)* 24(9), 1874-1883. doi: 10.1002/oby.21581.

Cava, E., Yeat, N.C., and Mittendorfer, B. (2017). Preserving Healthy Muscle during Weight Loss. *Adv Nutr* 8(3), 511-519. doi: 10.3945/an.116.014506.

Chennaoui, M., Desgorces, F., Drogou, C., Boudjemaa, B., Tomaszewski, A., Depiesse, F., et al. (2009). Effects of Ramadan fasting on physical performance and metabolic, hormonal, and inflammatory parameters in middle-distance runners. *Appl Physiol Nutr Metab* 34(4), 587-594. doi: 10.1139/H09-014.

Cho, A.R., Moon, J.Y., Kim, S., An, K.Y., Oh, M., Jeon, J.Y., et al. (2019). Effects of alternate day fasting and exercise on cholesterol metabolism in overweight or obese adults: A pilot randomized controlled trial. *Metabolism* 93, 52-60. doi: 10.1016/j.metabol.2019.01.002.

Chow, L.S., Manoogian, E.N.C., Alvear, A., Fleischer, J.G., Thor, H., Dietsche, K., et al. (2020). Time-Restricted Eating Effects on Body Composition and Metabolic Measures in Humans who are Overweight: A Feasibility Study. *Obesity (Silver Spring)* 28(5), 860-869. doi: 10.1002/oby.22756.

Cochran, W.G. (1954). The Combination of Estimates from Different Experiments. *Biometrics* 10(1), 101-129. doi: Doi 10.2307/3001666.

Cohen, J. (1992). A power primer. *Psychol Bull* 112(1), 155-159.

Davis, C.S., Clarke, R.E., Coulter, S.N., Rounsefell, K.N., Walker, R.E., Rauch, C.E., et al. (2016). Intermittent energy restriction and weight loss: a systematic review. *Eur J Clin Nutr* 70(3), 292-299. doi: 10.1038/ejcn.2015.195.

De Bock, K., Derave, W., Eijnde, B.O., Hesselink, M.K., Koninckx, E., Rose, A.J., et al. (2008). Effect of training in the fasted state on metabolic responses during exercise with carbohydrate intake. *J Appl Physiol (1985)* 104(4), 1045-1055. doi: 10.1152/japplphysiol.01195.2007.

Duval, S., and Tweedie, R. (2000). Trim and fill: a simple funnel-plot–based method of testing and adjusting for publication bias in meta-analysis. *Biometrics* 56(2), 455-463.

el Ati, J., Beji, C., and Danguir, J. (1995). Increased fat oxidation during Ramadan fasting in healthy women: an adaptative mechanism for body-weight maintenance. *Am J Clin Nutr* 62(2), 302-307. doi: 10.1093/ajcn/62.2.302.

Ferguson, L.M., Rossi, K.A., Ward, E., Jadwin, E., Miller, T.A., and Miller, W.C. (2009). Effects of caloric restriction and overnight fasting on cycling endurance

performance. *J Strength Cond Res* 23(2), 560-570. doi: 10.1519/JSC.0b013e31818f058b.

Gabel, K., Hoddy, K.K., Haggerty, N., Song, J., Kroeger, C.M., Trepanowski, J.F., et al. (2018). Effects of 8-hour time restricted feeding on body weight and metabolic disease risk factors in obese adults: A pilot study. *Nutr Healthy Aging* 4(4), 345-353. doi: 10.3233/NHA-170036.

Hajek, P., Myers, K., Dhanji, A.R., West, O., and McRobbie, H. (2012). Weight change during and after Ramadan fasting. *J Public Health* 34(3), 377-381. doi: 10.1093/pubmed/fdr087.

Haouari, M., Haouari-Oukerro, F., Sfaxi, A., Ben Rayana, M.C., Kaabachi, N., and Mbazaa, A. (2008). How Ramadan fasting affects caloric consumption, body weight, and circadian evolution of cortisol serum levels in young, healthy male volunteers. *Horm Metab Res* 40(8), 575-577. doi: 10.1055/s-2008-1065321.

Harder-Lauridsen, N.M., Rosenberg, A., Benatti, F.B., Damm, J.A., Thomsen, C., Mortensen, E.L., et al. (2017). Ramadan model of intermittent fasting for 28 d had no major effect on body composition, glucose metabolism, or cognitive functions in healthy lean men. *Nutrition* 37, 92-103. doi: 10.1016/j.nut.2016.12.015.

Harvie, M., and Howell, A. (2017). Potential benefits and harms of intermittent energy restriction and intermittent fasting amongst obese, overweight and normal weight subjects—a narrative review of human and animal evidence. *Behav Sci* 7(1), 4.

Harvie, M., Wright, C., Pegington, M., McMullan, D., Mitchell, E., Martin, B., et al. (2013). The effect of intermittent energy and carbohydrate restriction v. daily energy restriction on weight loss and metabolic disease risk markers in overweight women. *Br J Nutr* 110(8), 1534-1547. doi: 10.1017/S0007114513000792.

Harvie, M.N., Pegington, M., Mattson, M.P., Frystyk, J., Dillon, B., Evans, G., et al. (2011). The effects of intermittent or continuous energy restriction on weight loss and metabolic disease risk markers: a randomized trial in young overweight women. *Int J Obes (Lond)* 35(5), 714-727. doi: 10.1038/ijo.2010.171.

Headland, M.L., Clifton, P.M., and Keogh, J.B. (2019). Effect of intermittent compared to continuous energy restriction on weight loss and weight maintenance after 12 months in healthy overweight or obese adults. *Int J Obes* 43(10), 2028-2036.

Headland, M.L., Clifton, P.M., and Keogh, J.B. (2020). Impact of intermittent vs. continuous energy restriction on weight and cardiometabolic factors: a 12-month follow-up. *Int J Obes (Lond),* 1-7. doi: 10.1038/s41366-020-0525-7.

Heilbronn, L.K., Smith, S.R., Martin, C.K., Anton, S.D., and Ravussin, E. (2005). Alternate-day fasting in nonobese subjects: effects on body weight, body composition, and energy metabolism. *Am J Clin Nutr* 81(1), 69-73. doi: 10.1093/ajcn/81.1.69.

Higgins, J.P., Thompson, S.G., Deeks, J.J., and Altman, D.G. (2003). Measuring inconsistency in meta-analyses. *BMJ* 327(7414), 557-560. doi: 10.1136/bmj.327.7414.557.

Ismail, S., Shamsuddin, K., Latiff, K.A., Saad, H.A., Majid, L.A., and Othman, F.M. (2015). Voluntary Fasting to Control Post-Ramadan Weight Gain among Overweight and Obese Women. *Sultan Qaboos Univ Med J* 15(1), e98-e104.

Kalam, F., Gabel, K., Cienfuegos, S., Wiseman, E., Ezpeleta, M., Steward, M., et al. (2019). Alternate day fasting combined with a low-carbohydrate diet for weight loss, weight maintenance, and metabolic disease risk reduction. *Obes Sci Pract* 5(6), 531-539. doi: 10.1002/osp4.367.

Karli, U., Guvenc, A., Aslan, A., Hazir, T., and Acikada, C. (2007). Influence of Ramadan Fasting on Anaerobic Performance and Recovery Following Short time High Intensity Exercise. *J Sports Sci Med* 6(4), 490-497.

Keogh, J.B., Pedersen, E., Petersen, K.S., and Clifton, P.M. (2014). Effects of intermittent compared to continuous energy restriction on short-term weight loss and long-term weight loss maintenance. *Clin Obes* 4(3), 150-156. doi: 10.1111/cob.12052.

Klempel, M.C., Kroeger, C.M., and Varady, K.A. (2013). Alternate day fasting (ADF) with a high-fat diet produces similar weight loss and cardio-protection as ADF with a low-fat diet. *Metabolism* 62(1), 137-143.

Kocaaga, T., Tamer, K., Karli, U., and Yarar, H. (2019). Effects of Ramadan Fasting on Physical Activity Level and Body Composition in Young Males. *Int J Appl Exerc Physio* 8(3).

Kreitzman, S.N., Coxon, A.Y., and Szaz, K.F. (1992). Glycogen storage: illusions of easy weight loss, excessive weight regain, and distortions in estimates of body composition. *Am J Clin Nutr* 56(1 Suppl), 292S-293S. doi: 10.1093/ajcn/56.1.292S.

Lessan, N., Saadane, I., Alkaf, B., Hambly, C., Buckley, A.J., Finer, N., et al. (2018). The effects of Ramadan fasting on activity and energy expenditure. *Am J Clin Nutr* 107(1), 54-61. doi: 10.1093/ajcn/nqx016.

Mattson, M.P., Longo, V.D., and Harvie, M. (2017). Impact of intermittent fasting on health and disease processes. *Ageing Res Rev* 39, 46-58. doi: 10.1016/j.arr.2016.10.005.

Mirzaei, B., Rahmani-Nia, F., Moghadam, M.G., Ziyaolhagh, S.J., and Rezaei, A. (2012). The effect of ramadan fasting on biochemical and performance parameters in collegiate wrestlers. *Iran J Basic Med Sci* 15(6), 1215-1220.

Moher, D., Liberati, A., Tetzlaff, J., Altman, D.G., and Group, P. (2009). Reprint—preferred reporting items for systematic reviews and meta-analyses: the PRISMA statement. *Phys Ther* 89(9), 873-880.

Moro, T., Tinsley, G., Bianco, A., Marcolin, G., Pacelli, Q.F., Battaglia, G., et al. (2016). Effects of eight weeks of time-restricted feeding (16/8) on basal metabolism, maximal strength, body composition, inflammation, and cardiovascular risk factors in resistance-trained males. *J Transl Med* 14(1), 290. doi: 10.1186/s12967-016-1044-0.

Muhammad, H.F.L., Latifah, F.N., and Susilowati, R. (2018). The yo-yo effect of Ramadan fasting on overweight/obese individuals in Indonesian: A prospective study. *Med J Nutrition Metab* 11(2), 127-133. doi: 10.3233/mnm-17188.

Nachvak, S.M., Pasdar, Y., Pirsaheb, S., Darbandi, M., Niazi, P., Mostafai, R., et al. (2019). Effects of Ramadan on food intake, glucose homeostasis, lipid profiles and body composition composition. *Eur J Clin Nutr* 73(4), 594-600. doi: 10.1038/s41430-018-0189-8.

Naharudin, M.N.B., and Yusof, A. (2018). The effect of 10 days of intermittent fasting on Wingate anaerobic power and prolonged high-intensity time-to-exhaustion cycling performance. *Eur J Sport Sci* 18(5), 667-676. doi: 10.1080/17461391.2018.1438520.

Norouzy, A., Salehi, M., Philippou, E., Arabi, H., Shiva, F., Mehrnoosh, S., et al. (2013). Effect of fasting in Ramadan on body composition and nutritional intake: a prospective study. *J Hum Nutr Diet* 26 Suppl 1, 97-104. doi: 10.1111/jhn.12042.

Ramadan, J. (2002). Does fasting during Ramadan alter body composition, blood constituents and physical performance? *Med Princ Pract* 11 Suppl 2, 41-46. doi: 10.1159/000066413.

Ramadan, J., Telahoun, G., Al-Zaid, N.S., and Barac-Nieto, M. (1999). Responses to exercise, fluid, and energy balances during Ramadan in sedentary and active males. *Nutrition* 15(10), 735-739. doi: 10.1016/s0899-9007(99)00145-8.

Rohin, M.A., Rozano, N., Abd Hadi, N., Mat Nor, M.N., Abdullah, S., and Dandinasivara Venkateshaiah, M. (2013). Anthropometry and body composition status during Ramadan among higher institution learning centre staffs with different body weight status. *Sci World J*, 308041. doi: 10.1155/2013/308041.

Roy, A.S., and Bandyopadhyay, A. (2015). Effect of Ramadan intermittent fasting on selective fitness profile parameters in young untrained Muslim men. *BMJ Open Sport Exerc Med* 1(1), e000020. doi: 10.1136/bmjsem-2015-000020.

Salehi, M., and Neghab, M. (2007). Effects of fasting and a medium calorie balanced diet during the holy month Ramadan on weight, BMI and some blood parameters of overweight males. *Pak J Biol Sci* 10(6), 968-971.

Schoenfeld, B.J., Aragon, A.A., Wilborn, C.D., Krieger, J.W., and Sonmez, G.T. (2014). Body composition changes associated with fasted versus non-fasted aerobic exercise. *J Int Soc Sports Nutr* 11(1), 54. doi: 10.1186/s12970-014-0054-7.

Sedgwick, P., and Marston, L. (2013). Meta-analyses: standardised mean differences. *Bmj-Brit Med J* 347. doi: Artn F725710.1136/Bmj.F7257.

Stannard, S.R., and Thompson, M.W. (2008). The effect of participation in Ramadan on substrate selection during submaximal cycling exercise. *J Sci Med Sport* 11(5), 510-517. doi: 10.1016/j.jsams.2007.03.003.

Sterne, J.A., and Egger, M. (2001). Funnel plots for detecting bias in meta-analysis: guidelines on choice of axis. *J Clin Epidemiol* 54(10), 1046-1055. doi: 10.1016/s0895-4356(01)00377-8.

Stote, K.S., Baer, D.J., Spears, K., Paul, D.R., Harris, G.K., Rumpler, W.V., et al. (2007). A controlled trial of reduced meal frequency without caloric restriction in healthy, normal-weight, middle-aged adults. *Am J Clin Nutr* 85(4), 981-988.

SÜLÜ, B., ÖZtÜRk, B., GÜVen, A., and KiliÇ, K. (2010). The Effect of Long-Term Controlled Fasting (The Ramadan Model) on Body Mass Index, Blood

Biochemistry and Oxidative Stress Factors. *Turk Klin J Med Sci* 30(3), 855-863. doi: 10.5336/medsci.2008-9981.

Syam, A.F., Sobur, C.S., Abdullah, M., and Makmun, D. (2016). Ramadan fasting decreases body fat but not protein mass. *Int J Endocrinol Metab* 14(1).

Templeman, I., Thompson, D., Gonzalez, J., Walhin, J.P., Reeves, S., Rogers, P.J., et al. (2018). Intermittent fasting, energy balance and associated health outcomes in adults: study protocol for a randomised controlled trial. *Trials* 19(1), 86. doi: 10.1186/s13063-018-2451-8.

Teng, N.I., Shahar, S., Rajab, N.F., Manaf, Z.A., Johari, M.H., and Ngah, W.Z. (2013). Improvement of metabolic parameters in healthy older adult men following a fasting calorie restriction intervention. *Aging Male* 16(4), 177-183. doi: 10.3109/13685538.2013.832191.

Thomas, B.H., Ciliska, D., Dobbins, M., and Micucci, S. (2004). A process for systematically reviewing the literature: providing the research evidence for public health nursing interventions. *Worldviews Evid Based Nurs* 1(3), 176-184. doi: 10.1111/j.1524-475X.2004.04006.x.

Tinsley, G.M., Forsse, J.S., Butler, N.K., Paoli, A., Bane, A.A., La Bounty, P.M., et al. (2017). Time-restricted feeding in young men performing resistance training: A randomized controlled trial. *Eur J Sport Sci* 17(2), 200-207. doi: 10.1080/17461391.2016.1223173.

Tinsley, G.M., Moore, M.L., Graybeal, A.J., Paoli, A., Kim, Y., Gonzales, J.U., et al. (2019). Time-restricted feeding plus resistance training in active females: a randomized trial. *Am J Clin Nutr* 110(3), 628-640. doi: 10.1093/ajcn/nqz126.

Trabelsi, K., el Abed, K., Stannard, S.R., Jammoussi, K., Zeghal, K.M., and Hakim, A. (2012a). Effects of fed- versus fasted-state aerobic training during Ramadan on body composition and some metabolic parameters in physically active men. *Int J Sport Nutr Exerc Metab* 22(1), 11-18. doi: 10.1123/ijsnem.22.1.11.

Trabelsi, K., Stannard, S.R., Ghlissi, Z., Maughan, R.J., Kallel, C., Jamoussi, K., et al. (2013). Effect of fed- versus fasted state resistance training during Ramadan on body composition and selected metabolic parameters in bodybuilders. *J Int Soc Sports Nutr* 10(1), 23. doi: 10.1186/1550-2783-10-23.

Trabelsi, K., Stannard, S.R., Maughan, R.J., Jammoussi, K., Zeghal, K., and Hakim, A. (2012b). Effect of resistance training during Ramadan on body composition and

markers of renal function, metabolism, inflammation, and immunity in recreational bodybuilders. *Int J Sport Nutr Exerc Metab* 22(4), 267-275. doi: 10.1123/ijsnem.22.4.267.

Trepanowski, J.F., Kroeger, C.M., Barnosky, A., Klempel, M., Bhutani, S., Hoddy, K.K., et al. (2018). Effects of alternate-day fasting or daily calorie restriction on body composition, fat distribution, and circulating adipokines: Secondary analysis of a randomized controlled trial. *Clin Nutr* 37(6 Pt A), 1871-1878. doi: 10.1016/j.clnu.2017.11.018.

Trepanowski, J.F., Kroeger, C.M., Barnosky, A., Klempel, M.C., Bhutani, S., Hoddy, K.K., et al. (2017). Effect of alternate-day fasting on weight loss, weight maintenance, and cardioprotection among metabolically healthy obese adults: a randomized clinical trial. *JAMA Intern Med* 177(7), 930-938.

Van Proeyen, K., Szlufcik, K., Nielens, H., Pelgrim, K., Deldicque, L., Hesselink, M., et al. (2010). Training in the fasted state improves glucose tolerance during fat-rich diet. *J Physiol* 588(Pt 21), 4289-4302. doi: 10.1113/jphysiol.2010.196493.

Varady, K. (2011). Intermittent versus daily calorie restriction: which diet regimen is more effective for weight loss? *Obes Rev* 12(7), e593-e601.

Varady, K.A., Bhutani, S., Klempel, M.C., Kroeger, C.M., Trepanowski, J.F., Haus, J.M., et al. (2013). Alternate day fasting for weight loss in normal weight and overweight subjects: a randomized controlled trial. *Nutr J* 12(1), 146. doi: 10.1186/1475-2891-12-146.

Varady, K.A., Hoddy, K.K., Kroeger, C.M., Trepanowski, J.F., Klempel, M.C., Barnosky, A., et al. (2016). Determinants of weight loss success with alternate day fasting. *Obes Res Clin Pract* 10(4), 476-480. doi: 10.1016/j.orcp.2015.08.020.

Vargas, A.J., Pessoa, L.D., and da Rosa, R.L. (2018). Intermittent fasting and low carb diet in the body composition of women practicing physical activity. *Rev Bras Nutr Esportiva* 12(72), 483-490.

Widhalm, K., Pöppelmeyer, C., and Helk, O. (2017). The Effect of Alternate-Day Fasting (ADF) on Weight Loss, Metabolic Parameters and Psychological Characteristics. *Aktuelle Ernährungsmedizin* 42(03), 188-192. doi: 10.1055/s-0043-109126.

Ziaee, V., Razaei, M., Ahmadinejad, Z., Shaikh, H., Yousefi, R., Yarmohammadi, L., et al. (2006). The changes of metabolic profile and weight during Ramadan fasting. *Singapore Med J* 47(5), 409-414.

Zouhal, H., Saeidi, A., Salhi, A., Li, H., Essop, M.F., Laher, I., et al. (2020). Exercise Training and Fasting: Current Insights. *Open Access J Sports Med* 11, 1-28. doi: 10.2147/OAJSM.S224919.
